# Effect of Levels of Acetate on the Mevalonate Pathway of *Borrelia burgdorferi*

**DOI:** 10.1371/journal.pone.0038171

**Published:** 2012-05-31

**Authors:** Tricia A. Van Laar, Ying-Han Lin, Christine L. Miller, S. L. Rajasekhar Karna, James P. Chambers, J. Seshu

**Affiliations:** South Texas Center for Emerging Infectious Diseases and Department of Biology, Center for Excellence in Infection Genomics and The University of Texas at San Antonio, San Antonio, Texas, United States of America; University of Kentucky College of Medicine, United States of America

## Abstract

*Borrelia burgdorferi*, the agent of Lyme disease, is a spirochetal pathogen with limited metabolic capabilities that survives under highly disparate host-specific conditions. However, the borrelial genome encodes several proteins of the mevalonate pathway (MP) that utilizes acetyl-CoA as a substrate leading to intermediate metabolites critical for biogenesis of peptidoglycan and post-translational modifications of proteins. In this study, we analyzed the MP and contributions of acetate in modulation of adaptive responses in *B. burgdorferi*. Reverse-transcription PCR revealed that components of the MP are transcribed as individual open reading frames. Immunoblot analysis using monospecific sera confirmed synthesis of members of the MP in *B. burgdorferi*. The rate-limiting step of the MP is mediated by HMG-CoA reductase (HMGR) via conversion of HMG-CoA to mevalonate. Recombinant borrelial HMGR exhibited a *K_m_* value of 132 µM with a *V_max_* of 1.94 µmol NADPH oxidized minute^−1^ (mg protein)^−1^ and was inhibited by statins. Total protein lysates from two different infectious, clonal isolates of *B. burgdorferi* grown under conditions that mimicked fed-ticks (pH 6.8/37°C) exhibited increased levels of HMGR while other members of the MP were elevated under unfed-tick (pH 7.6/23°C) conditions. Increased extra-cellular acetate gave rise to elevated levels of MP proteins along with RpoS, CsrA_Bb_ and their respective regulons responsible for mediating vertebrate host-specific adaptation. Both lactone and acid forms of two different statins inhibited growth of *B. burgdorferi* strain B31, while overexpression of HMGR was able to partially overcome that inhibition. In summary, these studies on MP and contributions of acetate to host-specific adaptation have helped identify potential metabolic targets that can be manipulated to reduce the incidence of Lyme disease.

## Introduction

Lyme disease is the most prevalent arthropod-borne disease in the United States with nearly 30,000 cases reported to the Centers for Disease Control and Prevention (CDC) in 2010 [1]. The disease is caused by a spirochetal pathogen, *Borrelia burgdorferi*, following transmission from infected ticks belonging to the *Ixodes* spp [2], [3]. The incidence of Lyme disease is highest in geographic areas where there are increased ecological interactions between humans, reservoir hosts infected with *B. burgdorferi*, and transmission vectors [3]–[11]. Lyme disease is a multi-phasic disorder leading to severe arthritis, carditis, and/or neuroborreliosis if untreated during the early stages of infection [5]–[10]. While doxycycline is the antibiotic of choice for treatment during early stage Lyme disease, the efficacy of antibiotics can be variable in patients presenting with established, late-stage *B. burgdorferi* infection [12], [13]. Currently, no vaccines are available against Lyme disease in humans [14], necessitating development of alternate strategies to limit the transmission of *B. burgdorferi* to humans and thus reducing the incidence of Lyme disease.

*B. burgdorferi* is unique among bacteria since it is neither Gram-positive nor Gram-negative, as it does not possess the typical lipopolysaccharide (LPS) characteristic of Gram-negative bacteria [15]. The *B. burgdorferi* genome is rather unique in that several key metabolic pathways for synthesis of essential compounds such as amino acids, nucleic acids, and fatty acids are absent [16]. Thus, the bacterium is intimately associated with its host in order to acquire these required nutrients. The largest components of the outer membrane lipid mass of *B. burgdorferi* are two glycolipids identified as cholesteryl 6-*O-*acyl-β-D-galactopyranoside (BbGL-I; 23.2%) and 1,2-di-*O*-acyl-3-*O*-α-D-galactopyranosyl-*sn*-glycerol (BbGL-II; 12.4%) [17]–[20]. A third glycolipid, cholesteryl-β-D-galactopyranoside, also makes up a portion of the outer membrane. These glycolipids function in a manner similar to LPS in that they are important outer membrane components, surface exposed, and able to elicit antibody responses in mice [17]–[20]. The outer membrane also has free cholesterol and other cholesterol esters [20], suggesting that either biogenesis of acquisition of cholesterol is an important metabolic requirement for *B. burgdorferi.*

**Figure 1:**
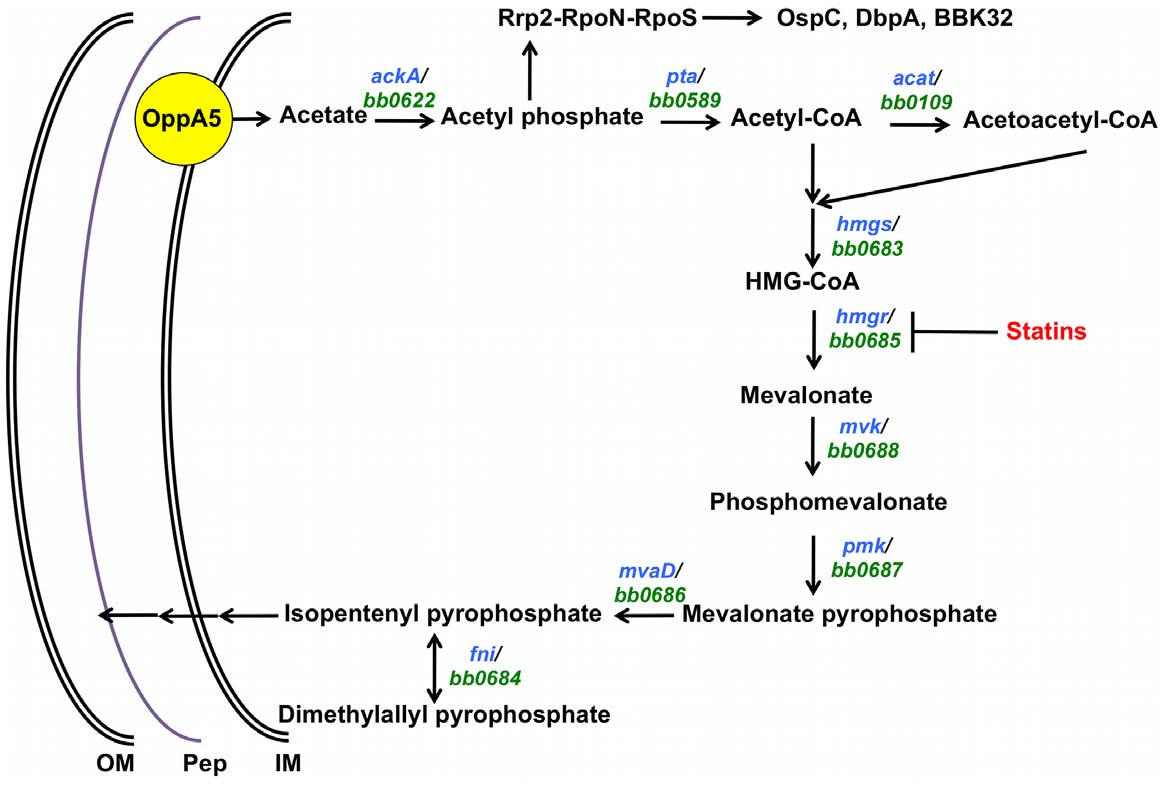
A model for the role of acetate in modulating the vertebrate host-specific adaptation and the mevalonate pathway in *B. burgdorferi*. Intracellular levels of acetate contribute to the activation of the Rrp2-RpoN-RpoS pathway leading to the adaptation of *B. burgdorferi* to vertebrate host-specific conditions and provide the initiating substrate for the mevalonate pathway. It appears that acetate plays a central role in modulating host-specific adaptation to central metabolic functions critical for survival of *B. burgdorferi* under different environmental conditions. The rate-limiting step of the mevalonate pathway, HMG-CoA reductase (HMGR), is a potential chemotherapeutic target that can be exploited to reduce the survival of *B. burgdorferi* and hence a reduction in the incidence of Lyme disease. Sequence analysis of members of the MP in *B. burgdorferi* indicated significant similarity with ORFs of the MP that have been characterized in *L. monocytogenes, P. mevalonii* and *S. aureus* (Table 4) [16]. Based on this analysis, *B. burgdorferi* appears to possess all open reading frames (ORFs *bb0683* to *bb0688*) necessary for the production of IPP via the mevalonate pathway [16].

Sequence analysis of the borrelial genome indicates the presence of homologs (*bb0683*-*bb0688*) of the mevalonate pathway (MP) leading to the synthesis of isopentenyl-5-pyrophosphate (IPP); however, there was a conspicuous lack of homologs that will lead to synthesis of cholesterol [16] (Fig. 1). IPP is an essential component of several isoprenoids and a precursor for peptidoglycan synthesis contributing to the structural integrity of several organisms [21]–[26]. The MP is responsible for synthesis of IPP in archaea, eukaryotes, and some eubacteria where mevalonate is a key intermediate of the pathway [27], [28]. The rate-limiting step of the MP in eukaryotes is the reduction of 3-hydroxy-3-methyl-glutaryl-coenzyme A (HMG-CoA) to mevalonate by HMG-CoA reductase (HMGR; EC 1.1.1.88) [21]–[23], [26], [29], [30]. In humans, HMGR is the target of HMGR inhibitors referred to as statins [31]–[33]. The inhibition of HMGR by statins is responsible for lowering levels of plasma low-density lipoproteins (LDL) leading to a reduction in several clinical manifestations that are directly or indirectly linked to elevated levels of LDL [30], [31], [33]–[35]. Moreover, due to the central role played by IPP in isoprenoid synthesis, organisms that do not possess MP have an alternate pathway for the synthesis of IPP [27]. The 2-*C*-methyl-D-erythritol 4-phosphate/1-deoxy-D-xylulose 5-phosphate (MEP/DOXP) pathway is found in most eubacteria and some plants where it is used in tandem with MP or exclusively to synthesize IPP [27], [28].

The absence of homologs leading to the synthesis of cholesterol from IPP suggests that *B. burgdorferi* most probably acquires cholesterol derivatives from its hosts and key intermediates of the MP are utilized for the biogenesis of peptidoglycan or for post-translational modification of proteins such as incorporation of prenyl groups. Hence, it appears that the MP could play a critical role in not only providing the key intermediates required for synthesis of peptidoglycan and/or the cell wall but also for borrelial surface determinants that mediate interactions of *B. burgdorferi* with its vertebrate or invertebrate hosts. Moreover, cholesterol and its derivatives could also play a role in the synthesis of glycolipids and post-transcriptional modifications in concert with MP contributing to the host-pathogen interactions of *B. burgdorferi.*

Recently, we and others have analyzed the role of Carbon Storage Regulator A of *B. burgdorferi* (CsrA_Bb_) in regulating the adaptation of *B. burgdorferi* for infection of the vertebrate host [6], [10], [36]–[38]. Sze and Li showed that CsrA_Bb_ represses phosphate acetyltransferase (Pta) leading to increased accumulation of acetyl phosphate which in turn leads to activation of the Rrp2-RpoN-RpoS pathway [37], [39]. A number of studies have shown that this pathway drives expression of a variety of determinants that facilitate transmission of *B. burgdorferi* from the tick vector and mediate interaction with vertebrate host-specific proteins [11], [36], [40]–[48]. Moreover, we have also shown that one of the 5 oligopeptide permeases containing a solute binding domain plays a role in the intracellular accumulation of acetate from the extracellular milieu affecting vertebrate host-specific adaptation [9]. Since the MP in *B. burgdorferi* is dependent on levels of acetyl-CoA, the extracellular concentrations of acetate, its transport from the extracellular environment, and conversion to acetyl-CoA via the enzymes acetate kinase (AckA) and phosphate acetyltransferase (Pta) [37], presumably play a role in the regulation of this pathway linking the host-specific modulation of *B. burgdorferi* and key metabolic pathways for cell wall biogenesis and post-translational modifications.

In this study, we characterize the MP and the role of levels of acetate in modulating the MP and vertebrate host-specific adaptation in *B. burgdorferi*. Using recombinant borrelial HMGR, we examined the kinetics of mevalonate formation and the inhibitory effects of select statins on growth of *B. burgdorferi*. Additionally, we determined differential expression of MP members under conditions mimicking the unfed- and fed-tick midgut and the concomitant effects of supplemental acetate on host-specific adaptation and levels of key components of the MP in *B. burgdorferi*. These studies will aid in identification and design of compounds that inhibit this central metabolic pathway that could potentially affect both the survival of *B. burgdorferi* but also its ability to exhibit its pathogenic effects in vertebrate hosts. Analysis of these potential inhibitory compounds should add to the array of strategies to reduce the incidence and debilitating effects of Lyme disease in endemic areas.

## Materials and Methods

### Bacterial Strains and Growth Conditions

Two clonal, infectious isolates of *B. burgdorferi* strain B31 (MSK5 and A3) and one non-infectious isolate (ML23) were used in this study [49], [50]. All *B. burgdorferi* cultures used for transcriptional analysis were grown in 1% CO_2_ at 32°C in BSK-II liquid medium (pH 7.6) supplemented with 6% normal rabbit serum (Pel-Freez Biologicals, Rogers, AR) [49], [51]. We propagated *B. burgdorferi* strain B31 (clonal isolates MSK5 and A3 that carry the full complement of plasmids needed for infection of the vertebrate host) to a density of 5×10^7^ spirochetes/ml in BSK-II growth medium. The tick mid-gut, before (unfed-tick, pH 7.6/23°C) and after (fed-tick, pH 6.8/37°C) a blood meal [5]–[10], was mimicked in order to determine if these environmental signals altered levels of proteins of the MP. *B. burgdorferi* strain B31-A3 was grown under fed-tick (pH 6.8/37°C), laboratory (pH 7.6/32°C), and unfed-tick (pH 7.6/23°C) specific conditions with varying concentrations of supplemental sodium acetate (0, 15, 30, and 90 mM) [9], [38]. The spirochetes were grown to a density of 5×10^7^ cells/ml and washed in Hanks Balanced Salt Solution (HBSS, Thermo Scientific HyClone, Logan, UT) three times to remove BSK-II growth medium and processed further for analysis of proteins. *Escherichia coli* TOP10 (Invitrogen, Carlsbad, CA), M15 (QIAgen, Valencia, CA), and Rosettagami (Novagen) strains were used for all procedures involving cloning and overexpression of recombinant proteins, respectively. The *E. coli* strains were cultured in Luria-Bertani (LB) broth supplemented with appropriate concentrations of antibiotics [5].

### Transcriptional Analysis of ORFs of the MP

In order to determine if the ORFs of the MP of *B. burgdorferi* were being transcribed under *in vitro* growth conditions, total RNA was extracted as previously described [6]. Briefly, *B. burgdorferi* cultures were propagated at pH 7.6/32°C to a density of 2–3×10^7^ spirochetes per ml. The samples were washed in HBSS and the bacterial pellets resuspended in RNA-Bee (Tel-Test, Inc. Friendswood, TX) at a ratio of 0.2 ml for every 10^6^ cells. Following extraction with chloroform, the RNA was precipitated with isopropanol, washed with 75% ethanol, air-dried, and resuspended in RNAse-free water. The RNA was treated twice at 37°C for 45 minutes with DNaseI to remove any contaminating DNA and total RNA quantified spectrophotometrically. In order to evaluate sample RNA purity, real-time PCR was carried out using *recA* primers (recAFq and recARq) to detect contaminating DNA [6]. Samples devoid of contaminating DNA were reverse transcribed to cDNA using TaqMan reverse transcription reagents (Applied Biosystems, Foster City, CA) [10]. The cDNA was PCR amplified using primers (Table 1) specific to the internal regions of MP members *bb0683*-*bb0688*.

**Table 1:** Oligonucleotides used in this study.

Name	Sequence (5′→3′)^a^
BB0589F/NdeI	ACGCCATATGCAAAAGTTAAAGGGAGTTACGAAG
BB0589R/XhoI	ACGCCTCGAGTTAAATGCTTATCATTAAAGCACTT
BB0622F/NdeI	ACGCCATATGACAGAAAGATTTGAAAAAGG
BB0622R/XhoI	ACGCCTCGAGTTTATTTAAAATTTCTGAGGATC
BB0683F/NdeI	AGCGCATATGAGAATAGGTATTAGTGATATTAG
BB0683R/XhoI	ACGCCTCGAGGGCTCGATACCCATAAACTCGG
BB0685F/BamHI	ACGCGGATCCATGAACTTGGAGTCTTTAAGC
BB0685R/SalI	ACGCGTCGACTTAAAGCTAAGGTTGCATGAACT
BB0686F/NdeI	ACGCCATATGAAAATAAAGTGTAAAGTT
BB0686R/XhoI	ACGCCTCGAGAATCCATTCTAAGTCACA
BB0687F/NdeI	ACGCCATATGGATTTGATTAGTTTTTCT
BB0687R/XhoI	ACGCCTCGAGGCATTTATCGCTTTC
BB0688F/NdeI	ACGCCATATGCTAAGAATAAGAAAGCCT
BB0688R/XhoI	ACGCCTCGAGAGTCTCAATTACCTT
BB0683cDNAF	ACGCAACTTTCCAGGTTCAACATGCG
BB0683cDNAR	ACGCTCCATAGCAGCTTCAACTCCATC
BB0684cDNAF	ACGCATGACAGGGGGCAGTAAAGAGG
BB0684cDNAR	ACGCCCAAGGCTGAACAATTCCTTAACG
BB0685cDNAF	ACGCGAATCTTCTGTTGTTGCTGCCC
BB0685cDNAR	ACGCGCTCGCTCCTCTTCGTAAAAACC
BB0686cDNAF	ACGCTTCCCGACAGCAGCAGGCC
BB0686cDNAR	ACGCTCCCCTCATTTAGCAAATCAGCAG
BB0687cDNAF	ACGCGTTTGCTTACTTGAGTCAAAATTG
BB0687cDNAR	ACGCTTTCATCTCCAAATTGCATTTC
BB0688cDNAF	ACGCTTTCTGAAATTCCTATTGGAGTTG
BB0688cDNAR	ACGCTCCTTATTCTGAAAAGAAGCATAAG
PflacF/BamHIKpnI	ACGCGGATCCGGTACCGACGTCTAATACCGAGCTTCA
PflacR/EcoRV	ACGCGATATCCATTGGAAACCTCCCTCAATTG
BB0685R/SalIKpnI	ACGCCTCGAGGGTACCGTCGACTTAAAGCTAAGGTTG

a Restriction enzyme sites are underlined.

### Cloning, Over-expression and Purification of Recombinant Proteins

Total genomic DNA obtained from *B. burgdorferi* clonal isolate MSK5 was used as template to PCR amplify *pta* (*bb0589*), *ackA* (*bb0622*), *hmgs* (*bb0683*), *hmgr* (*bb0685*), *mvaD* (*bb0686*), *pmk* (*bb0687*), or *mvk* (*bb0688*) using forward and reverse primers containing appropriate engineered restriction enzyme sites (Table 1). The amplicons were cloned into pCR2.1-TOPO vector (Invitrogen) and transformed into *E. coli* TOP 10 cells and subjected to blue/white colony screen in the presence of ampicillin (100 µg/ml) and kanamycin (50 µg/ml). Respective inserts were excised with appropriate restriction enzymes (Table 1) and ligated into either the pMAL-p2X (New England Biolabs, Ipswich, MA) facilitating expression of recombinant protein with a N-terminal maltose binding protein (MBP) or pET23a (Novagen) expression vector for expression of recombinant proteins with a C-terminal 6X Histidine tag (Table 2). Plasmid pET45b/*Lmhmgr* used for purification of the listerial homolog of HMGR (LmHMGR) with an N-terminal 6X Histidine tag was provided by JA Friesen [23]. Ligated products were electro-transformed into *E. coli* TOP 10 cells and screened by restriction enzyme digestion for the presence of inserts of appropriate size. The junctions of plasmids containing inserts of expected size were confirmed by sequence analysis and these plasmids were used to electro-transform the *E. coli* expression host (Rosettagami or M15). Transformants were plated on LB agar containing ampicillin (100 µg/ml) and kanamycin (25 µg/ml) (pMAL-p2X-*hmgr*) or LB agar containing ampicillin (100 µg/ml), chloramphenicol (34 µg/ml), kanamycin (15 µg/ml), and tetracycline (12.5 µg/ml) for the following constructs: pET23a/*pta*, pET23a/*ackA*, pET23a/*hmgs*, pET23a/*mvaD,* pET23a/*pmk,* pET23a/*mvk*, pET45b/*Lmhmgr*. Colonies were selected, grown overnight at 37°C in 5 mL of LB containing antibiotics, and transferred to one liter LB containing antibiotics and 0.2% glucose and allowed to grow until reaching an optical density of 0.5 at 600 nm. Expression of recombinant proteins was induced by addition of 1 mM isopropylthiogalactopyranoside (IPTG). For expression of recombinant HMGR-MBP, the cells were allowed to grow for 16 hrs at 16°C. Cells were harvested by centrifugation at 5000×*g* for 10 min and resuspended in 20 ml low-salt buffer (LSB; 10 mM phosphate buffer, pH 7.0 containing 30 mM NaCl and 10 mM β-mercaptoethanol). Bacterial pellets were disrupted using a French press and cell lysate was centrifuged at 8500×*g* for 20 min. Supernatant was loaded onto an amylose column (New England Biolabs) and the protein was purified using the Biologic Duoflow Chromatography system (BioRad). The column was washed with buffer A (LSB) and protein eluted with Buffer B (LSB with 50 mM maltose). For expression of HMGS, MvaD, Pmk, Mvk, Pta, AckA and LmHMGR, cells were allowed to grow for 4 hrs at 37°C, harvested by centrifugation at 5000 x *g* for 10 min and resuspended in 5 ml lysis buffer. Bacterial pellets were disrupted (French press) and the cell lysate centrifuged at 8500×*g* for 20 min. HMGS, MvaD, Pmk, Mvk, Pta and AckA were purified to homogeneity using the S&S Elutrap electro-separation method (Schleicher and Schull, Keene, NH) according to manufacturer’s instructions. LmHMGR was subjected to affinity purification using Ni-nitrilotriacetic acid (NiNTA) beads (QIAgen) per manufacturer's instructions. Bound histidine-tagged proteins were eluted as 0.5 ml fractions with elution buffer (250 mM imidazole). Purified proteins were analyzed by SDS-12.5% polyacrylamide gel electrophoresis. Purified recombinant proteins were quantified using the BCA protein assay (Pierce, Thermo Fisher Scientific, Rockford, IL) and stored at −80°C until further use.

**Table 2:** Plasmids used in this study.

Plasmid	Description	Source or reference
pCR®2.1-TOPO	PCR cloning vector	Invitrogen
pMAL-p2x	Expression vector with an N-terminal maltose binding protein tag	New England Biolabs
pET23a	Expression vector with a C-terminal 6-His tag	Novagen
pTV146	*bb0685* cloned into pCR2.1 for protein expression	This study
pTV147	*bb0685* cloned into pMAL-p2x	This study
pTV118	*bb0686* cloned into pCR2.1	This study
pTV125	*bb0686* cloned into pET23a	This study
pTV119	*bb0687* cloned into pCR2.1	This study
pTV101	*bb0687* cloned into pET23a	This study
pTV120	*bb0688* cloned into pCR2.1	This study
pTV121	*bb0688* cloned into pET23a	This study
pTV131	*bb0683* cloned into pET23a	This study
pET45b/*lmhmgr*	*Lmhmgr* cloned into pET45b	[23]
pYL03	*bb0589* cloned into pET23a	This study
pYE04	*bb0622* cloned into pET23a	This study
pCR2.1/P*_flac_*	P*_flac_* cloned into pCR2.1	Unpublished data, D. Scott Samuels
pTV142	P*_flac_* cloned into pCR2.1 with KpnI and NheI restriction sites	This study
pTV145	*bb0685* cloned into pCR2.1 with NheI and EcoRV/KpnI restriction sites	This study
pTV146	P*_flac_/bb0685* cloned into pCR2.1 with flanking KpnI sites	This study
pBVS2/*lacI*	Borrelial shuttle vector with kan^r^ and *lacI* under the control of P*_flgB_*	[55], [56]
pTR1	pBVS2/*lacI* with P*_flac_/bb0685*	This study

### Enzyme Assays

Recombinant *B. burgdorferi* HMGR enzyme activity was assayed spectrophotometrically following the oxidation of NADPH to NADP^+^ at 340 nm. The reaction buffer consisted of 200 mM KCl, 50 mM Tris-HCl, 1 mg/ml BSA, 0.17 mM HMG-CoA, 0.13 mM NADPH, and 0.5 µg enzyme at pH 7.5 in a final reaction volume of 200 µl [23]. The reduction in absorbance at 340 nm was monitored at 5 sec intervals over a course of 30 sec the resulting velocity was used to obtain a Lineweaver-Burk Plot (not shown). Nonlinear regression using GraphPad Prism 4.0 software was used to calculate the *K*_m_ and *V*_max_. Purified human HMGR (Sigma-Aldrich, St. Louis, MO) and LmHMGR were used as positive assay controls (data not shown). An additional control included addition of purified maltose binding protein (MBP) alone to the reaction buffer and enzyme assays performed as described above. For inhibition assays, the reaction mixture was supplemented with either lovastatin or simvastatin at a concentration of 250 µM [23] or an equivalent volume of diluent (DMSO) control and the enzyme assay was performed as described above.

### Generation of Anti-serum

Purified recombinant proteins (HMGR, MvaD, Pmk, Mvk, Pta and AckA) were emulsified in equal volumes of Titermax™ (Sigma, St Louis MO) and used to immunize 6 to 8 week old female BALB/c mice. Booster immunizations were given at days 14 and 21 [5]–[10]. Immunoblot analysis was carried out to determine the specificity of the antibodies against the recombinant proteins (data not shown) and total borrelial lysates with serum obtained on day 28 post-immunization. All animal procedures were conducted in accordance with an approved animal use protocol from the Institutional Animal Care and Use Committee of The University of Texas at San Antonio.

### Sodium Dodecyl Sulfate-polyacrylamide Gel Electrophoresis (SDS-PAGE) and Immunoblot Analysis

Whole-cell *B. burgdorferi* lysates were prepared and separated on SDS-12.5% PAGE as previously described [5]–[10]. Separated proteins were either visualized by Coomassie brilliant blue staining or transferred onto a PVDF membrane (Amersham Hybond™-P; GE Healthcare, Buckinghamshire, UK) and subjected to immunoblot analysis as previously described [5], [7], [8], [10]. Membranes were probed with α-OspA, α-HMGR, α-MvaD, α-Pmk, α-Mvk, α-BBA34, α-BBK32, α-DbpA, α-OspC, α-Pta, α-P66, α-BBA64, α-BosR, α-OppA1, α-OppA2, α-OppA3, α-OppA4, α-RpoS, α-FlaB, α-NapA, and α-AckA antibodies. Blots were incubated with appropriate concentrations of HRP conjugated anti-mouse, anti-rat, or anti-rabbit secondary antibodies and developed using ECL™ Western blotting detection reagents (GE Healthcare).

### Statin Activation

Simvastatin (Sigma-Aldrich, St. Louis, MO) was activated as described by Robinzon, et al [52]. Briefly, 4 mg simvastatin was resuspended in 150 µl 100% EtOH to which 150 µl 0.1 N NaOH was added and the mixture incubated at 50°C for 2 hrs. The pH was brought to 7.0 with HCl and the final volume was brought to 1 ml with ddH_2_O. Lovastatin (Sigma-Aldrich, St. Louis, MO) was activated as described by Ghosh, et al [53]. Briefly, 5 mg lovastatin was resuspended in 100 µl 100% EtOH to which 50 µl 0.6 M NaOH was added, and the mixture was incubated at 50°C for 2 hrs. Following incubation, the pH was brought to 7.5 with 0.4 M HCl and the final volume of the mixture was brought to 1 ml with ddH_2_O. Statins in the inactivated form were resuspended in DMSO to a final concentration of 20 mg/ml.

### Effect of Statins on *In vitro* Growth of *B. burgdorferi*

Cultures of a clonal infectious isolate of *B. burgdorferi* strain B31-A3 were grown to a density of 5×10^7^ cells per mL in BSK-II media supplemented with 6% NRS at pH 7.6/32°C. Cells were pelleted by centrifugation (4000×*g*, 20 min at 4°C) and washed three times with HBSS supplemented with 0.2% glucose. After washing, cells were plated on 96-well plates containing various dilutions (2.5–320 µg/ml) of statins, in both acid and lactone forms [54]. The cells were treated with statins for 2 hrs at 32°C. Following treatment, cells were washed three times with HBSS +0.2% glucose. After washing, cells were resuspended in BSK-II media with 6% NRS and allowed to grow for 3 weeks at 32°C in 96-well plates. Growth was scored at three weeks using darkfield microscopy. All data are averages of three independent assays. Data were subjected to unpaired Student's *t* test implemented in Excel software. Asterisks indicate values that are statistically significantly different between control and treated samples (***, *P*<0.001).

### Effect of Statins on Viability of *B. burgdorferi*

*B. burgdorferi* strain B31-A3 was washed three times with HBSS +0.2% glucose and treated with 2.5, 10, 20, 40 or 160 µg/ml of statins for 2 hrs. After washing to remove free drug, the viability of spirochetes was evaluated using the Live/Dead *BacLight* bacterial viability kit (Molecular Probes, Invitrogen, Carlsbad, CA) in conjunction with confocal microscopy [5], [10]. Images were captured using a Zeiss LSM510 microscope and deconvolved using AutoQuantX (MediaCybernetics Inc., Bethesda, MD).

### HMGR Overexpression Construct

Total genomic DNA obtained from *B. burgdorferi* clonal isolate MSK5 (Table 1) was used as template to PCR amplify *hmgr* (*bb0685*) while a plasmid designated pCR2.1/P*_flac_* (unpublished data, D. Scott Samuels) was used as template to PCR amplify P*_flac_* using forward and reverse primers in order to obtain appropriate engineered restriction enzyme sites for subsequent cloning steps (Table 1). The amplicons were cloned into pCR2.1-TOPO vector (Invitrogen) and transformed into *E. coli* TOP 10 cells and subjected to blue/white colony screen in the presence of ampicillin (100 µg/ml) and kanamycin (50 µg/ml). P*_flac_* was excised with appropriate restriction enzymes (Table 1) and ligated into pCR2.1/*hmgr*. The resulting P*_flac_*/*hmgr* was excised with KpnI and ligated into pBSV2/lacI [55], [56], giving a plasmid designated pTR1, in which the borrelial *hmgr* was placed under the control of the IPTG-inducible P*_flac_* promoter.

### Generation of Borrelial HMGR Overexpression Strain

A clonal derivative of *B. burgdorferi* sensu stricto strain B31 lacking lp25, ML23, was electrotransformed with pTR1 using a procedure described previously [57], [58]. After electroporation, the transformants were incubated for 48 h at 32°C in BSK-II growth medium without antibiotics and plated on BSK-II agarose overlays containing 200 µg/ml of kanamycin. The plates were incubated at 32°C in 1% CO_2_ for 21 days or until individual colonies were visible in the overlays. Colonies were isolated aseptically into BSK-II growth medium with 200 µg/ml of kanamycin until the density reached 5×10^7^ spirochetes/ml. One ml of culture was used to extract total genomic DNA and the presence of the plasmid was confirmed using primers specific to the *lacI* gene. Three transformants were identified as being positive for *lacI*, and one of those clones, designated TR1 (Table 3) was used for further study.

**Table 3:** *Borrelia burgdorferi* strains used in this study.

B. burgdorferi strain	Description	Source or reference
A3	B31 isolate with all infection-associated plasmids capable of transformation	[50], [71]
MSK5	B31 isolate with all infection-associated plasmids	[49]
ML23	B31 isolate lacking lp-25; non-infectious	[49]
TR1	ML23 transformed with pTR1	This study

### Induction of HMGR Expression in the Overexpression Strain, TR1

TR1 was grown to a density of 5×10^7^ spirochetes/ml at which time IPTG was added to a final concentration of 5 mM and analyzed for expression of HMGR by immunoblot analysis. After 16 hrs of induction with IPTG, the cells were treated with statins as described above.

## Results

### Sequence Analysis of the Members of Mevalonate Pathway (MP)

**Figure 2:**
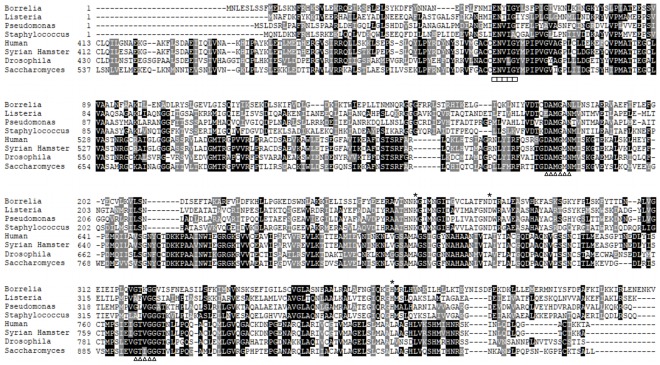
Sequence comparison of HMGR homologs. ClustalX alignment of HMGR from eight different species, representing both Class II (*Borrelia burgdorferi, Listeria monocytogenes, Pseudomonas aeruginosa, Staphylococcus aureus*) and Class I (Human, Syrian Hamster, *Drosophila melanogaster*, and *Saccharomyces cerevisiae*) shows a high degree of similarity with regard to binding sites for HMG-CoA/HMG-CoA reductase inhibitors (ENVIG - boxes) and NAD(P)H (DAMGXN and GTVGG - triangles). Indicated by stars are conserved residues shown to be important for catalysis of HMGR in *Pseudomonas mevalonii* [29].

The contributions of CsrA_Bb_ in regulating virulence gene expression and adaptation of *B. burgdorferi* to vertebrate host have been recently reported [6], [10], [37], [38]. A key component of this regulation is the intracellular levels of acetate. Acetate is phosphorylated to acetyl-phosphate by the enzyme acetate kinase (AckA; BB0622). The conversion of acetyl-phosphate to acetyl-CoA by phosphate acetyltransferase (Pta, BB0589) appears to be a critical switch in mediating the relay of signals for activation of the linear gene regulatory network, Rrp2-RpoN-RpoS, resulting in the expression of determinants facilitating the adaptation of *B. burgdorferi* to vertebrate host. A previous report [39] and additional bio-informatic analysis for potential pathways dependent on levels of acetyl-CoA revealed that the borrelial genome contains several ORFs with significant sequence similarity to members of the MP in other organisms (Fig 2; Table 4). The initiating substrate of the MP is acetyl-CoA, which is converted to acetoacetyl-CoA by acetoacetyl transferase (ACAT; BB0109). Both acetyl-CoA and acetoacetyl-CoA are used to generate HMG-CoA by the action of HMG-CoA synthase (HMGS; BB0683). The conversion of HMG-CoA to mevalonate by HMG-CoA reductase (HMGR; BB0685) has been shown to be the rate-limiting step of the MP [59]. The presence of ORFs with sequence similarity to mevalonate kinase (Mvk; BB0688), phosphomevalonate kinase (Pmk; BB0687) and mevalonate decarboxylase (MvaD; BB0686) to homologs present in other bacteria and the conspicuous absence of members of the non-mevalonate pathway suggest that isopentenyl pyrophosphate, a central metabolic intermediate for cell wall biogenesis and post-translational modification of proteins, is generated in *B. burgdorferi* via the MP.

**Table 4:** Sequence comparison of *B. burgdorferi* mevalonate pathway proteins to characterized homologs in other bacterial species.

Species [ref]	*B. burgdorferi*[this study]	*L. monocytogenes* [23]	*S. aureus* [26]	*P. mevalonii* [22]	*H. volcanii* [72]	SyrianHamster [73]	Human [74]
*K*_m_	132±22	19.8±1.2	40	20	60	20	4.4±0.7
*V*_max_	1.92±0.16	18.2±0.5	21	66	34	64	188±35

### *bb0685* Encodes a Class II HMGR

Genomes of organisms classified under each of the three domains of life are known to carry homologs of HMGR [60]. While Class I homologs of HMGR characterized by the presence of an N-terminal membrane-anchoring domain are found in eukaryotes and some archaea, the Class II homologs lacking this N-terminal domain are present in some eubacteria and archaea [21]. Both classes have a catalytic domain for the binding of HMG-CoA [61]. Sequence alignment of three eubacterial (Class II; *Listeria monocytogenes, Pseudomonas mevalonii,* and *Staphylococcus aureus*) and four eukaryotic (Class I; *Homo sapiens, Mesocricetus auratus, Drosophila melanogaster,* and *Saccharomyces cerevisiae*) HMGR sequences with that of *B. burgdorferi* (BB0685) revealed high degree of similarity in the binding sites for HMG-CoA and NAD(P)H (Fig. 2), indicating that borrelial HMGR has the ability to catalyze the conversion of HMG-CoA to mevalonate and this pathway could link the levels of acetate to the end-products of MP. The borrelial homolog has ENYIG as the residues at the potential HMG-CoA binding site (sequence underscored by squares, Fig. 2) while other homologs have residues EN(T/V)(I/L)G. The first region for NAD(P)H binding (sequence underscored by triangles, Fig. 2), is conserved among all eight species (DAMGAN for Class II and DAMGMN for Class I) while there was variation in the second binding region. Three or four of the five residues of the *B. burgdorferi* HMGR binding site are conserved when compared to the HMGRs of other species. The borrelial homolog also has conserved residues (indicated by asterisks, Fig. 2) that have been implicated in catalysis in *Pseudomonas mevalonii* HMGR [29], the canonical Class II HMGR. These observations, in conjunction with enzyme kinetics described below indicate that *bb0685* in *B. burgdorferi* encodes a Class II HMGR.

### HMGR from *B. burgdorferi* Exhibits Enzyme Activity

**Figure 3:**
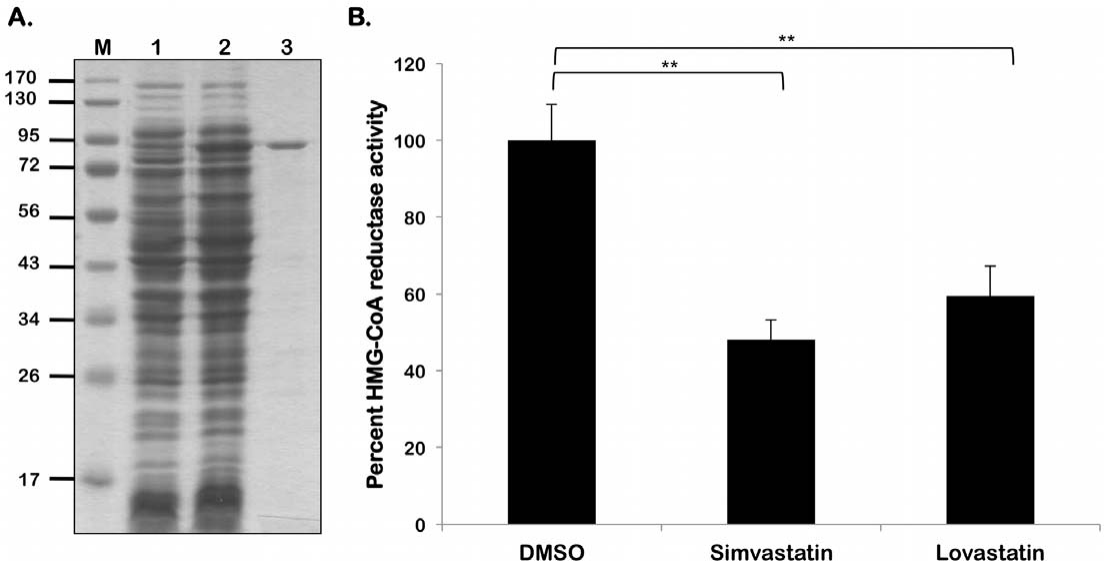
Statins inhibit the enzyme activity of *B. burgdorferi* HMGR. (A) Lysates purified HMGR as indicated above the respective lanes were resolved by SDS-12.5%PAGE. Gels were stained with Coomassie blue. Recombinant HMGR of *B. burgdorferi* purified by FPLC. Lane 1, non-induced *E. coli* containing pMAL-p2X/*hmgr*. Lane 2, *E. coli* containing pMAL-p2X/*hmgr* induced with 1 mM IPTG. Lane 3, Purified MBP-HMGR fusion protein. (B) Recombinant *B. burgdorferi* HMGR activity was measures as described under Materials and Methods. Statins were added to the enzyme reaction at a concentration of 250 µM. The velocity of the control reaction was calculated as 1.41±0.10 µmol NADPH oxidized minute^−1^ (mg protein)^−1^, set as 100% maximal activity in the presence of the statin diluent, DMSO, alone_._ Simvastatin lowered HMGR activity to 48.2% of maximal (velocity of 0.87±0.08 µmol NADPH oxidized minute^−1^ (mg protein)^−1^), while lovastatin reduced HMGR activity to 61.4% of maximal activity (velocity of 0.68±0.07 µmol NADPH oxidized minute^−1^ (mg protein)^−1^). Data shown are the average of three independent assays. Asterisks indicate samples whose values are statistically significantly different between control and treated conditions (**, *P*<0.01).

As shown in Fig 3A, the borrelial homolog of HMGR was purified as an MBP-fusion protein and was used to for enzyme kinetic analysis. Purified *B. burgdorferi* HMGR was tested for its ability to convert HMG-CoA into mevalonate. The reaction was monitored spectrophotometrically at 340 nm following decreasing absorbance as NADPH was oxidized NADP^+^. The calculated *K_m_* for *B. burgdorferi* HMGR is 132 µM. When compared to other Class II HMGR *K_m_* values, the *B. burgdorferi* HMGR has a *K_m_* value between 3- and 8-fold higher (Table 5). The calculated *V_max_* of *B. burgdorferi* HMGR is 1.92 µmol NADPH oxidized minute^−1^ (mg protein)^−1^. Consistent with lower substrate affinity, the calculated *V_max_* of *B. burgdorferi* is lower than that of other HMG-CoA reductases.

**Table 5:** Kinetic values for HMG-CoA reductases of different species^a^.

*B. burgdorferi* Protein	Enzyme name	% Identity(*L. monocytogenes*/*S. aureus*)	% Similarity (*L. monocytogenes*/*S. aureus*)
AckA/BB0622	Acetate kinase	44/43	66/65
Pta/BB0589	Phosphate acetyltransferase	44/45	65/67
ACAT/BB0109	Acetyl-CoA acetyltransferase	36/35	57/55
HMGS/BB0683	HMG-CoA synthase	29/29	49/48
HMGR/BB0685	HMG-CoA reductase	34/34	53/54
Mvk/BB0688	Mevalonate kinase	28/28	47/48
Pmk/BB0687	Phosphomevalonate kinase	22/21	39/42
MvaD/BB0686	Phosphomevalonate decarboxylase	34/31	54/51
Fni/BB0684	Isopentenyl-diphosphate isomerase	30/28	50/53

a *K*_m_ and *V*_max_ for *B. burgdorferi* were derived from the experiments described in Materials and Methods and are the average of three independent replicates. *K*_m_ is in units of µM and *V*_max_ is in units of µmol NADPH oxidized minute^−1^ (mg protein)^−1^_._ *L. monocytogenes* (*Listeria monocytogenes*), *S. aureus* (*Staphylococcus aureus*), *P.* *mevalonii* (*Pseudomonas mevalonii*), *H. volcanii* (*Haloferax volcanii*).

### Statins Inhibit *B. burgdorferi* HMGR

We further examined if the HMGR activity of the borrelial homolog can be inhibited with specific, clinically used, HMG-CoA reductase inhibitors, i.e., statins. As shown in Fig 3B, both lovastatin and simvastatin were inhibitory to *B. burgdorferi* HMGR at a concentration of 250 µM [22]. The velocity of the control reaction was calculated as 1.41±0.10 µmol NADPH oxidized minute^−1^ (mg protein)^−1^_._ Lovastatin reduced HMGR activity to 61.4% of maximal activity (velocity of 0.68±0.07 µmol NADPH oxidized minute^−1^ (mg protein)^−1^), while simvastatin lowered HMGR activity to 48.2% of maximal (velocity of 0.87±0.08 µmol NADPH oxidized minute^−1^ (mg protein)^−1^). These observations suggest that if the *B. burgdorferi* HMGR homolog plays a central role in the MP, *B. burgdorferi* HMGR could be a useful target enzyme for inhibitory agents such as statins.

### Transcriptional Analysis of ORFs of the MP in *B.* *burgdorferi*

**Figure 4:**
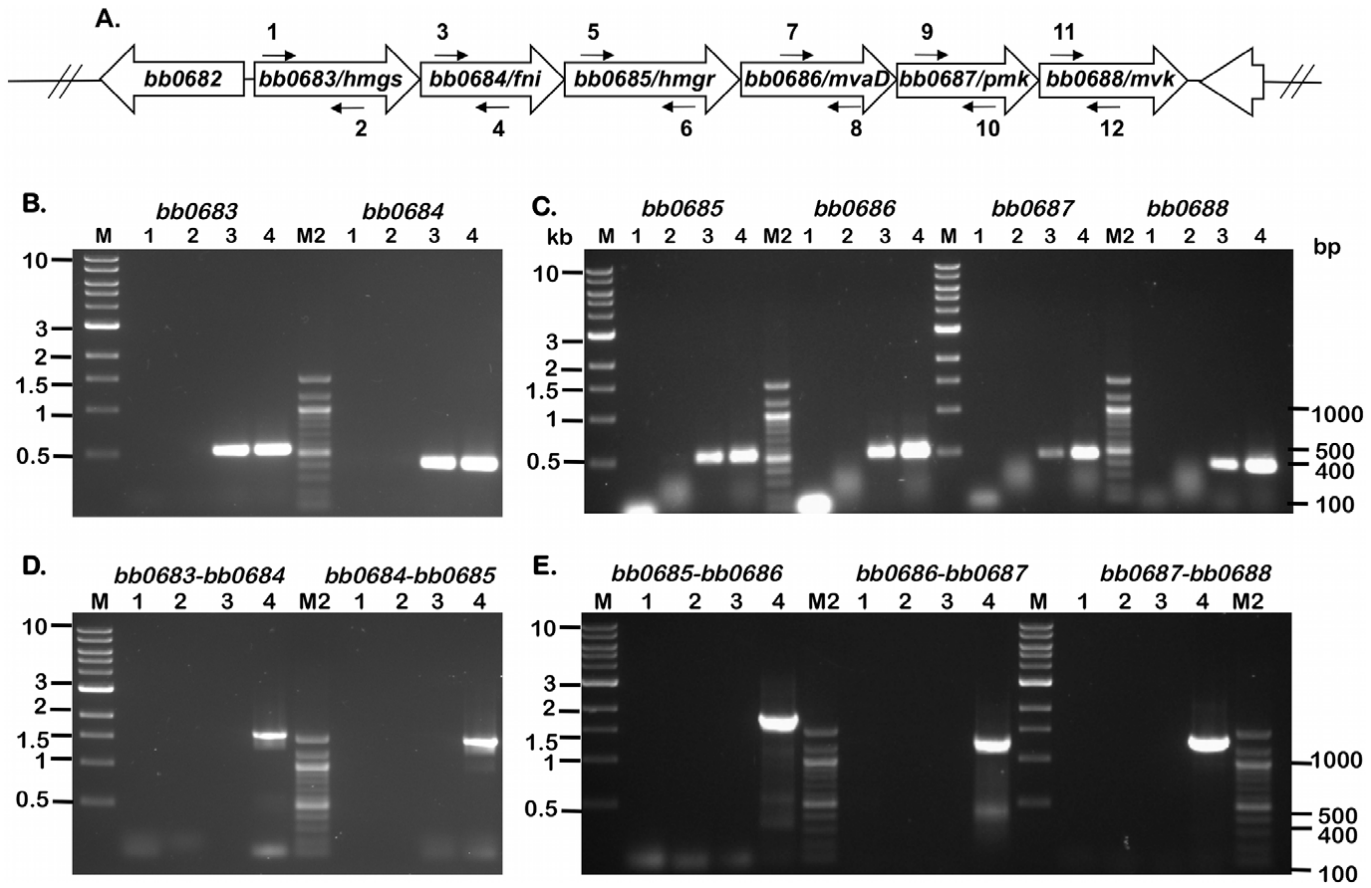
ORFs encoding members of the MP are transcribed in *B.* *burgdorferi* . (A) Schematic representation of the borrelial mevalonate pathway that extends from *bb0683* to *bb0688*. The arrows with numbers refer to primers used for the RT-PCR amplicons (depicted in B-E) separated on a 1% agarose gel stained with ethidium bromide. (B-E) The templates used in PCR amplification (B-E) are from *B. burgdorferi* strain B31 clonal isolate MSK5 and are as follows: Lane 1, PCR master mix with no template (double-distilled H_2_O control); Lane 2, total RNA (-RT control); Lane 3, cDNA (+RT); Lane 4, total genomic DNA. Lanes M and M2, molecular size markers in kilobases (M) or base pairs (M2) as indicated on the left and right sides, respectively. (B) Primers specific for *bb0683* (1 and 2) and *bb0684* (3 and 4) amplified cDNA (lane 3) and genomic DNA (lane 4). (C) Primers specific for *bb0685* (5 and 6), *bb0686* (7 and 8), *bb0687* (9 and 10) and *bb0688* (11 and 12) amplified cDNA (lane 3), and genomic DNA (lane 4) indicating active transcription of these ORFs. (D) Primers specific for the overlapping regions *bb0683-bb0684* (1 and 4), *bb0684-bb0685* (3 and 6) amplified genomic DNA (lane 4), but not cDNA (lane 3) indicating that the ORFs are not co-transcribed. (E) Primers specific for the overlapping regions *bb0685-bb0686* (5 and 8), *bb0686-bb0687* (7 and 10) and *bb0687-bb0688* (9 and 12) amplified genomic DNA (lane 4) but not cDNA (lane 3), indicating that the ORFs are not co-transcribed The images were generated using the Versadoc imaging system (Bio-Rad Laboratories, Hercules, CA).

In order to validate the *in silico* prediction of the presence of ORFs and determine the significance of the contributions of the members of the MP in the patho-physiology of *B. burgdorferi*, we carried out RT-PCR using primers specific to internal regions of the putative genes (Fig. 4A). As shown in Fig. 4B and 4C (Lane 3), when the primers specific to *bb0683*, *bb0684*, *bb0685*, *bb0686*, *bb0687* and *bb0688* were used with cDNA generated from total RNA extracted from MSK5, there was amplification of an approximate 400 bp fragment consistent with the amplicons size observed with MSK5 genomic DNA amplification (Fig. 4B; 4C, Lane 4). No amplification was observed when double distilled water (ddH_2_O) or total RNA (-RT) were used as PCR templates in the PCR mix (Fig. 4B, 4C, Lanes 1 and 2 respectively). We then determined if the genes of the mevalonate pathway were co-transcribed (Fig. 4D and 4E). No amplification was observed when primer sets corresponding to adjacent ORFs were used with cDNA generated from total MSK5 RNA (Fig. 4D, 4E, Lane 3) while amplification of appropriate product was observed when total MSK5 genomic DNA was used as template (Fig. 4D, 4E, Lane 4). As expected, no amplification occurred when ddH_2_O or total RNA (–RT) were used as controls (Fig. 4D, 4E, Lanes 1 and 2, respectively). Taken together, these observations indicate that genes of the mevalonate pathway are transcribed under *in vitro* growth conditions employed. However, the MP genes are not being co-transcribed as members of an operon (Fig. 4D, 4E).

### Levels of Expression of Members of the MP in *B.* *burgdorferi*

**Figure 5:**
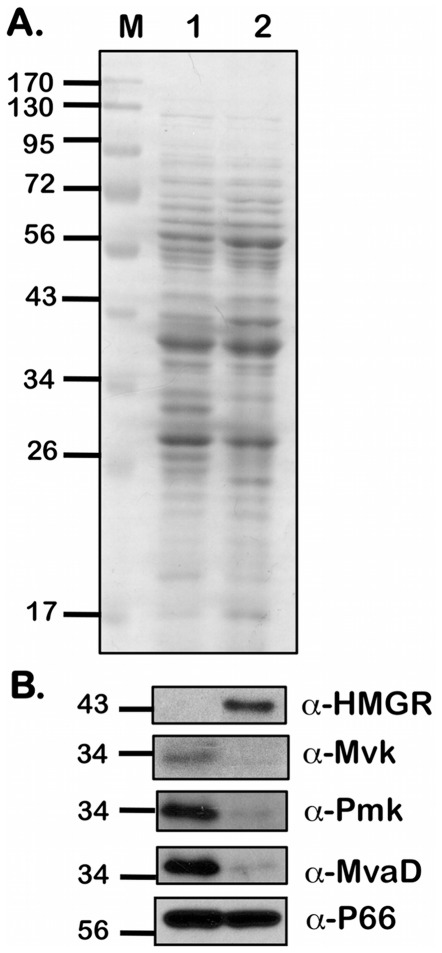
Levels of proteins of the MP in *B. burgdorferi* . Equivalent number of spirochetes from *B. burgdorferi* strain B31-A3 propagated in BSK-II medium with 6% NRS under conditions that either mimicked the unfed-tick (pH 7.6/23°C; Lane 1) or fed-tick (pH 6.8/37°C; Lane 2) to a density of 5 × 10^7^ spirochetes/ml were resolved by SDS-12.5%PAGE. Gels were stained with Coomassie blue (A) or separated proteins were electrotransfered onto PVDF membranes. (B) Immunoblots were incubated with mouse serum against purified HMGR, MvaD, Pmk, Mvk and P66 respectively. Blots were developed using the Enhanced Chemiluminescence system. Numbers to the left of the panels indicate the molecular mass standards in kilodaltons proximate to each of the antigens. Higher levels of HMGR expression were seen under fed-tick conditions when compared to unfed-tick conditions while the other three proteins showed higher levels of expression under unfed-tick conditions when compared to fed-tick conditions.

In order to determine if the transcriptional levels of members of the MP are reflected at the translational level, we determined the protein levels of a few members of the MP using mono-specific serum generated using recombinant proteins. There was increased expression of HMGR in an infectious clonal isolate of *B. burgdorferi* (B31-A3) that was propagated under conditions that mimicked fed ticks (pH 6.8/37°C; Fig. 5B, Lane 2, α-HMGR) when compared to that of unfed conditions (pH 7.6/23°C; Fig 5B, Lane 1, α-HMGR). However, the levels of Mvk, Pmk, and MvaD were elevated to varying degrees under conditions that mimicked unfed ticks (Fig 5B, Lane 1, α-Mvk, α-Pmk α-MvaD). The levels of these enzymes are therefore partly dependent on the host specific external signals such as temperature and pH which are known to facilitate *B. burgdorferi* adaptation to either the tick vector or the vertebrate host [62]–[66]. These observations suggest that the level of the rate-limiting enzyme HMGR is up-regulated under environmental conditions that mimic the vertebrate host and hence could be a rational target for inhibition to alter the adaptation of *B. burgdorferi*.

### Effect of Acetate on Protein Expression Profiles in *B.* *burgdorferi*

**Figure 6:**
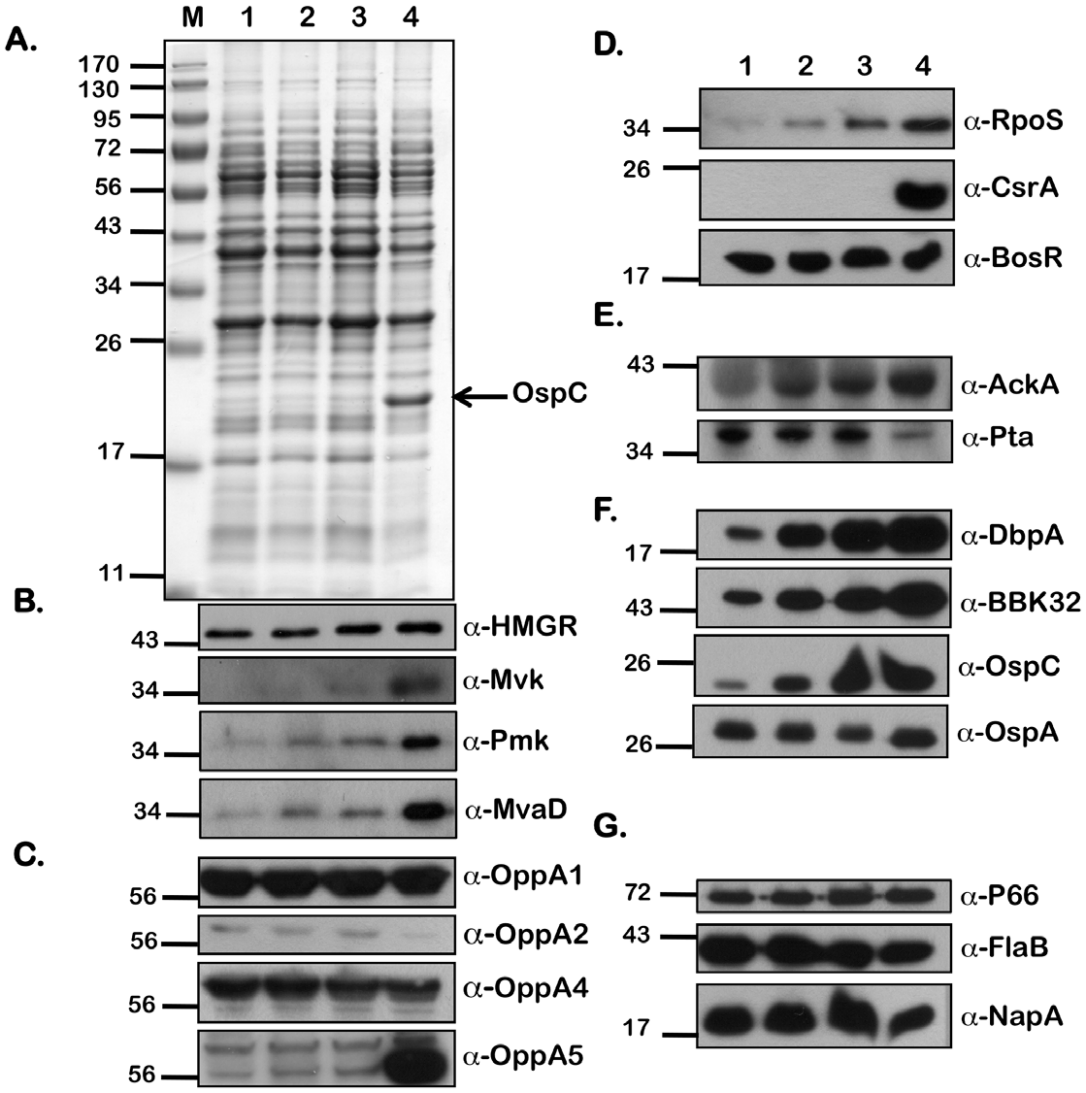
Effect of increasing concentrations of sodium acetate on levels of borrelial proteins under conditions that mimic fed-ticks. Equivalent numbers of spirochetes from *B. burgdorferi* B31-A3 propagated in BSK-II medium with 6% NRS under conditions that mimicked the fed-tick (pH 6.8/37°C) with increasing concentrations of supplemental NaOAc (from 0 mM - 90 mM) were resolved by SDS-12.5%PAGE. The gels were stained with Coomassie blue (A) or the separated proteins were electrotransfered onto PVDF membranes. Immunoblots (B-G) were probed with anti-serum against the antigens listed to the right of the blots. Blots were developed using the Enhanced Chemiluminescence system. The numbers to the left of the panels indicate the molecular mass standards in kilodaltons proximate to each of the antigens. Increased levels were seen with increasing concentrations of NaOAc for all proteins of the mevalonate pathway (B); OppA5 (C); gene regulators RpoS and CsrA_Bb_ (D); AckA (E); pathogenesis-related proteins DbpA, BBK32, and OspC (F). Decreased levels of protein expression were seen with OppA2 (C), Pta (E), and FlaB (G). Acetate levels had no effect on the other proteins tested.

**Figure 7:**
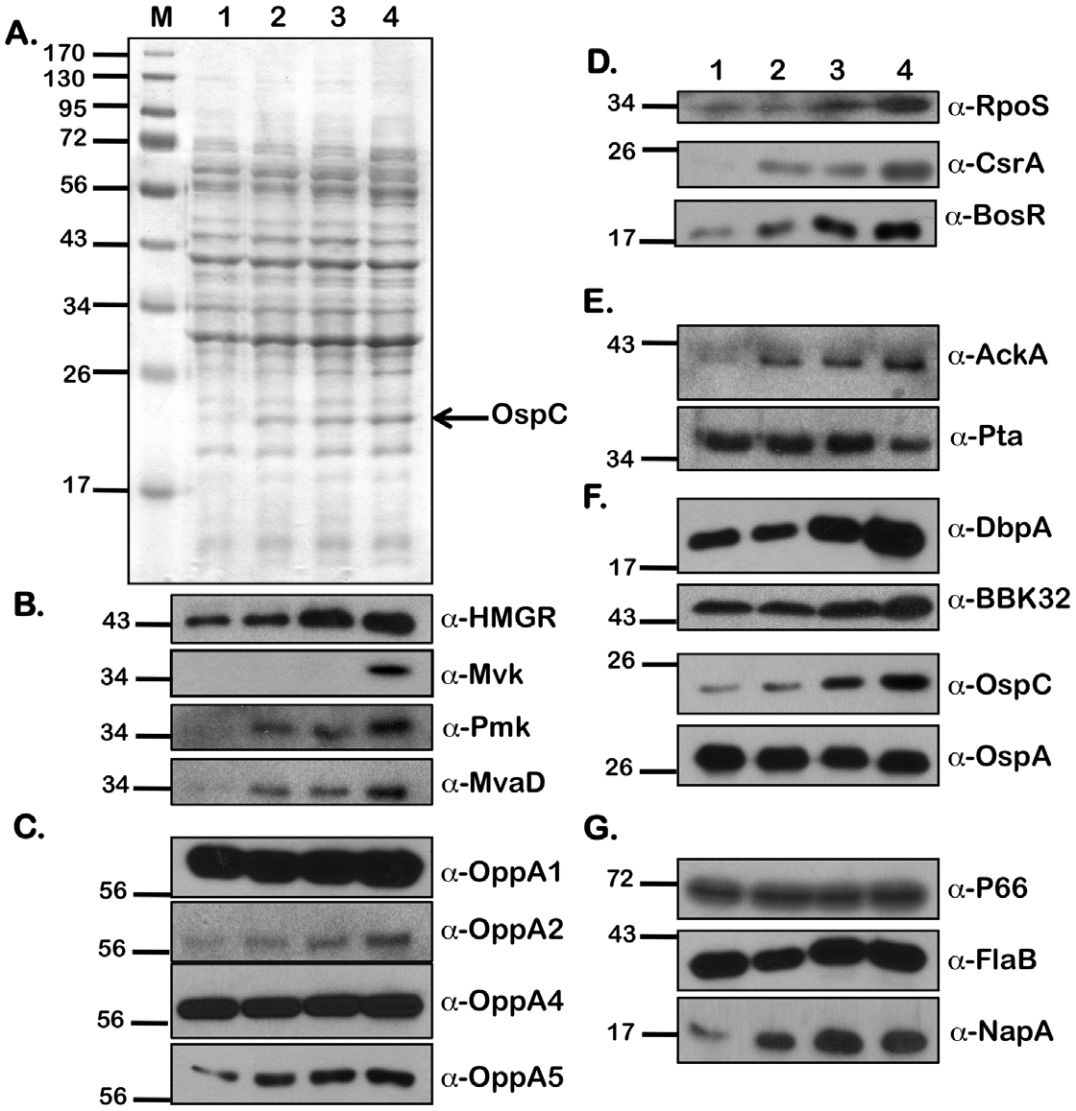
Effect of increasing concentrations of sodium acetate on levels of borrelial proteins under laboratory growth conditions. Equivalent numbers of spirochetes from *B. burgdorferi* B31-A3 propagated in BSK-II medium with 6% NRS at laboratory growth conditions (pH 7.6/32°C) with increasing concentrations of supplemental NaOAc (from 0 mM –90 mM) were resolved by SDS-12.5%PAGE. The gels were stained with Coomassie blue (A) or the separated proteins were electrotransfered onto PVDF membranes. Immunoblots (B-G) were probed with anti-serum against the antigens listed to the right of the blots. Blots were developed using the Enhanced Chemiluminescence system. Numbers to the left of the panels indicate the molecular mass standards in kilodaltons proximate to each of the antigens. Increased levels of protein expression were seen with increasing concentrations of NaOAc for all proteins of the mevalonate pathway (B); OppA5 (C); gene regulators RpoS, CsrA_Bb_, and BosR (D); AckA (E); pathogenesis-related proteins DbpA, BBK32, and OspC (F); NapA (G). Decreased levels of protein expression were seen with Pta (E). Acetate levels had no effect on the other proteins tested.

**Figure 8:**
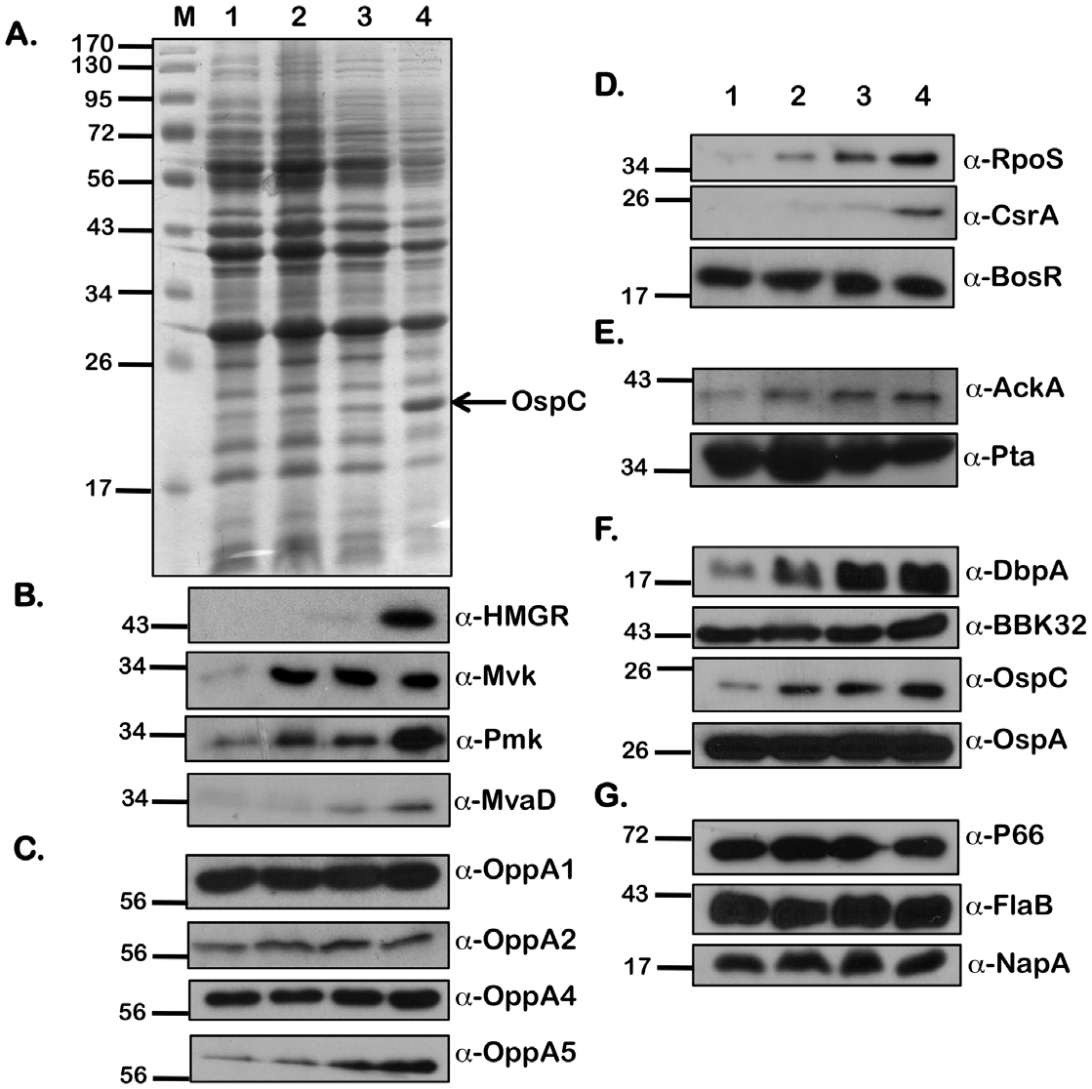
Effect of increasing concentrations of sodium acetate on levels of borrelial proteins under conditions that mimic unfed ticks. Equivalent numbers of spirochetes from A3 propagated in BSK-II medium with 6% NRS under conditions that mimicked the unfed-tick (pH 7.6/23°C) with increasing concentrations of supplemental NaOAc (from 0 mM –90 mM) were resolved by SDS-12.5%PAGE. The gels were stained with Coomassie blue (A) or the separated proteins were electrotransfered onto PVDF membranes. Immunoblots (B-G) were probed with anti-serum against the antigens listed to the right of the blots. Blots were developed using the Enhanced Chemiluminescence system. Numbers to the left of the panels indicate the molecular mass standards in kilodaltons proximate to each of the antigens. Increased levels of protein expression were seen with increasing concentrations of NaOAc for all proteins of the mevalonate pathway (B); OppA4, OppA5 (C), gene regulators RpoS and CsrA_Bb_ (D); AckA (E); pathogenesis-related proteins DbpA, BBK32, and OspC (F). Decreased levels of protein expression were seen with Pta (E). Acetate levels had no effect on the other proteins tested.

In addition to pH and temperature, we observed that increased levels of extracellular acetate could modulate vertebrate host-specific adaptation [9]. Since acetyl-CoA is the initial substrate of the MP, we hypothesized that the levels of acetyl-CoA could alter levels of the members of the MP in *B. burgdorferi*. We therefore determined levels of a variety of determinants that were altered in response to borrelial culture conditions with increased supplementation of acetate. Whole cell lysates from *B. burgdorferi* (A3) were propagated under fed-tick (pH 6.8/37°C), laboratory (pH 7.6/32°C), and unfed-tick (pH 7.6/32°C) growth conditions (Figs. 6, 7, and 8, respectively) with increasing levels of supplemental acetate (0, 15, 30, and 90 mM), separated by SDS-12.5% PAGE electrophoresis and stained with Coomassie Brilliant Blue (Figs. 6A, 7A, and 8A) or transferred to PVDF membranes for immunoblot analysis (Figs. 6B-F, 7B-F, and 8B-F). Consistent with previous observations [9], there was an increase in the level of OspC with increasing acetate, indicating increased levels of acetate were sufficient to increase the levels of OspC, even under temperature and pH conditions normally associated with unfed ticks (pH 7.6/23°C; Fig 7A). An increase in levels of HMGR, Mvk, Pmk and MvaD in *B. burgdorferi* propagated in media with increased levels of acetate (90 mM) was observed under fed (pH 6.8/37°C, Fig 6B), unfed (pH 7.6/23°C, Fig 8B) or laboratory growth conditions (pH 7.6/32°C, Fig 7B). Previously we noted that there were increased levels of OppA5 under pH/temperature mimicking fed-tick conditions (with no supplemental acetate) and were able to also show this increase in OppA5 with supplemental acetate independent of the temperature and pH (Figs 6B, 7B and 8B; α-OppA5). The levels of OppA1, OppA2, and OppA4 appeared to be constitutive and did not change following variation in the level of acetate or other environmental conditions such as temperature and pH (Figs. 6B and 8B; α-OppA1, α-OppA2, α-OppA4), with the exception of an increase seen in the levels of OppA2 under laboratory growth conditions (Fig. 7B; α-OppA2).

In order to further validate the effects of added acetate on the adaptation of *B. burgdorferi* to host-specific conditions, we determined levels of key regulators of gene expression as well as the select determinants increased in response to alterations in levels of borrelial regulators. Both RpoS and CsrA_Bb_ were increased in response to increasing levels of acetate under fed-tick, laboratory, or unfed-tick specific growth conditions (Figs. 6D, 7D, 8D; α-RpoS, α-CsrA_Bb_). While the level of BosR under laboratory growth conditions was increased in response to supplemental acetate (Fig 7D; α-BosR), no change in the level of BosR was observed under fed- or unfed tick conditions, (Fig. 6D, 8D; α-BosR). Additionally, the level of acetate kinase (AckA), the first key enzyme that modifies acetate to acetyl-phosphate, was elevated with increasing levels of supplemental acetate (Figs. 6E, 7E, 8E; α-AckA). Consistent with previous reports that CsrA_Bb_ acts to repress phosphate acetyl transferase (*pta*), we observed that levels of Pta were lower with increasing levels of acetate and coincided with increased levels of CsrA_Bb_. Many lipoproteins critical for infection of the mammalian host, i.e., DbpA, BBK32, and OspC were elevated with the increased RpoS and CsrA_Bb_ with increased added acetate under temperature and pH conditions mimicking the fed-tick, laboratory, or unfed tick (Figs. 6F, 7F, 8F; α-DbpA, α-BBK32, α-OspC). The only lipoprotein that did not show a significant difference was OspA (Figs. 6F, 7F, 8F; α-OspA). In addition to OspA, the levels of several other determinants such as 1) P66, an outer membrane protein that functions as a porin, 2) the major flagellin FlaB, and 3) a protein implicated in oxidative stress response, NapA, remained unchanged with increasing concentrations of acetate under different host-specific growth conditions tested (Figs. 6G, 7G, 8G; α-P66 α-FlaB and α-NapA). In summary, increasing acetate concentrations indicated that the MP in *B. burgdorferi* is responsive to levels of the initiating substrate acetyl-CoA. Moreover, the levels of regulators and lipoproteins indicative of adaptation of *B. burgdorferi* to conditions that mimic the fed tick or vertebrate host were all elevated in response to increasing levels of acetate and this effect was similar under different host-specific temperature or pH.

### Effect of Statins on Growth of *B. burgdorferi*

**Figure 9:**
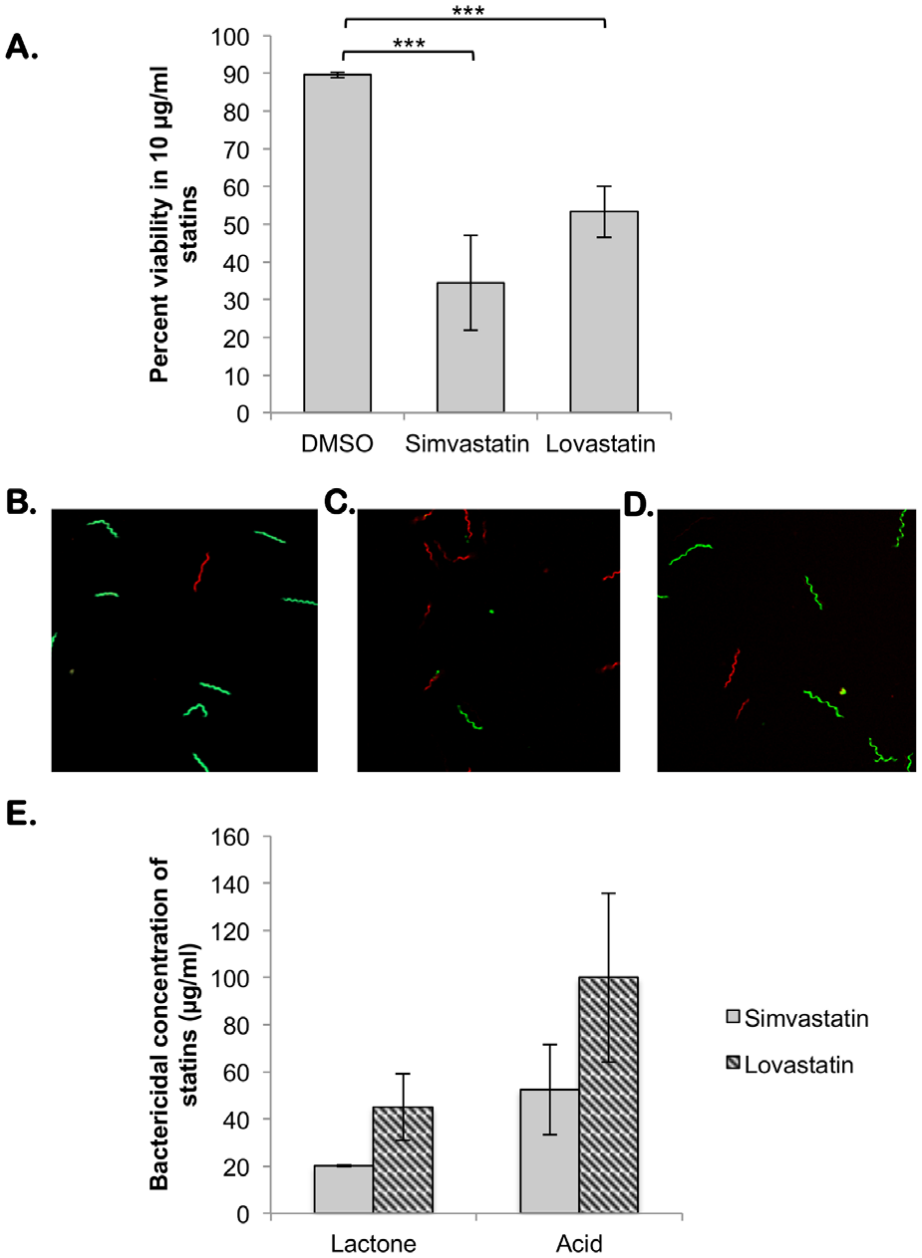
Sensitivity of *B. burgdorferi* to statins in the lactone or acid form. The spirochetes were treated with statins as described under Materials and Methods and analyzed for sensitivity by different methods. (A) Percent viability of *B. burgdorferi* treated with 10 µg/ml of simvastatin and lovastatin as determined by live/dead staining in conjunction with confocal microscopy. The percent viability is significantly reduced in statin treated bacteria when compared to cells treated with an equivalent volume of DMSO. (B-D) Confocal microscopy of live/dead stained *B. burgdorferi* following treatment with statins for two hours. Cells treated with DMSO (B) are green indicating live cells while those treated with 10 µg/ml of simvastatin (C) or lovastatin (D) are a mixture of green and red, indicating cell death. (E) Data shown are the average of three independent assays; bars indicate the standard deviation. Between 20 and 100 µg/ml simvastatin and lovastatin in either the acid (grey bars) or lactone (black bars) form inhibit growth of *B. burgdorferi in vitro* while the DMSO control appears to have no detrimental effect.

Based on the ability of select statins to inhibit recombinant borrelial HMGR, we evaluated the sensitivity of an infectious clonal isolate of *B. burgdorferi* strain B31-A3 with lovastatin and simvastatin either in the lactone (inactive) or acid (active) form prepared as described under Materials and Methods. As shown in Fig 9, DMSO (diluent) treated spirochetes were mostly alive with 89.6% viability (Fig 9A-B; green) under these experimental conditions while cells treated with 10 µg/ml of simvastatin or lovastatin showed significantly decreased viability (34.5% and 53.3%, respectively) and a mixture of live and dead (green/red; Fig. 9C-D) when analyzed by confocal microscopy. Treated spirochetes were resuspended in BSK-II media and grown for three weeks at 32°C. Growth was observed after three weeks using dark field microscopy and by change in the color of culture wells. The lactone form of simvastatin had a bactericidal concentration of 20 µg/ml while the lactone form of lovastatin had a bactericidal concentration of 45 µg/ml. The bactericidal concentration of the acid forms of simvastatin and lovastatin were 52.5 and 100 µg/ml, respectively. Simvastatin was a more potent inhibitor of borrelial growth under the specific conditions tested (Fig. 9D) compared to lovastatin. Both forms of the drugs inhibited spirochetal growth significantly (*P*<0.001) compared to that of spirochetes treated with the diluent or vehicle alone.

**Figure 10:**
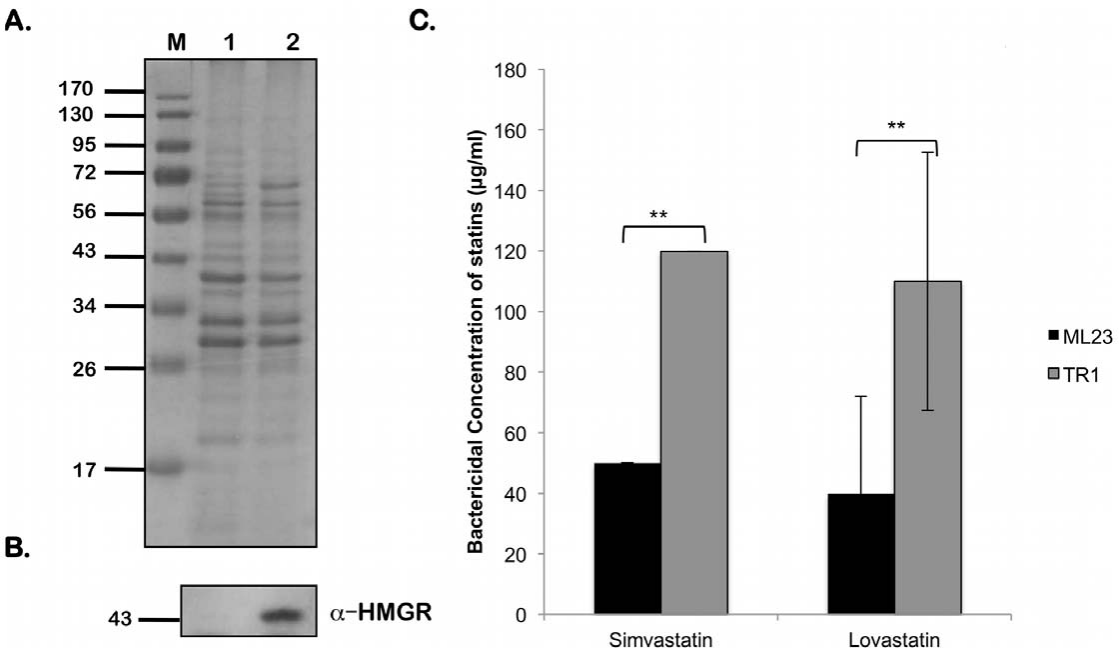
Sensitivity of the HMGR overexpression strain, TR1, to statins. Equivalent numbers of spirochetes from *B. burgdorferi* ML23 or TR1 propagated in BSK-II medium with 6% NRS at laboratory growth conditions (pH 7.6/32°C) treated with 5 mM IPTG for 16 hrs to induce HMGR expression as described under Materials and Methods and were resolved by SDS-12.5%PAGE. The gels were stained with Coomassie blue (A) or the separated proteins were electrotransfered onto PVDF membranes. Immunoblot (B) was probed with anti-serum against HMGR. Blots were developed using the Enhanced Chemiluminescence system. Numbers to the left of the panels indicate the molecular mass standards in kilodaltons. (C) Spirochetes were analyzed for sensitivity to increasing concentrations of stains. Data shown are the average of three independent assays; bars indicate the standard deviation. Data were subjected to the unpaired Student's *t* test implemented in Excel software. Asterisks indicate samples whose values are statistically significantly different between control and overexpression strains (**, *P*<0.01).

To indicate the importance of HMGR as the target of statins, we used the HMGR overexpression strain of *B. burgdorferi*, TR1, to determine whether the increased presence of HMGR would confer an increased resistance to statin treatment. As shown in Figure 10, TR1 has a significantly increased resistance to simvastatin and lovastatin (*P*<0.01) when compared to resistance of the parent strain, ML23.

Taken together, these observations demonstrate that statins can inhibit the activity of *B. burgdorferi* HMGR and subsequent growth of the bacteria, and the growth inhibition can be partially overcome with the overexpression of HMGR in *B. burgdorferi*. Thus it is important to characterize further the inhibitory effects of statins such that their use can possibly be extended to reduce the transmission of these spirochetes between reservoir hosts, ticks and dead-end vertebrate hosts.

## Discussion

The genome of *B. burgdorferi* encodes for limited metabolic capabilities which brings into focus the relevance of pathways that are apparently intact [16]. One such pathway is the mevalonate pathway (MP) that contains ORFs with significant identity to homologs in other bacterial systems indicating the importance of isopentenyl-5-pyrophosphate (IPP) - a key metabolic intermediate of this pathway. Since several metabolic processes, notably peptidoglycan biosynthesis and cell wall formation are dependent on the synthesis of IPP, pathways that lead to the synthesis of IPP are found across all three domains of life [27], [28]. Most organisms, including *B. burgdorferi,* possess the MP for IPP biosynthesis while others synthesize IPP through the more recently identified MEP/DOXP pathway [16]. We focused on the biochemical characterization of the MP, as it would expand our understanding of the nature of interactions between *B. burgdorferi* and its vertebrate or tick host. Moreover, the availability of a wide variety of statins exhibiting different pharmacokinetic properties to inhibit the rate-limiting enzyme (HMGR) of the MP in *B. burgdorferi* opens an avenue to evaluate an array of compounds potentially capable of preventing the transmission, colonization, and/or dissemination of the bacteria to vertebrate hosts [30], [31], [35].

In addition to identifying putative targets for inhibition, the MP is dependent on levels of acetyl-CoA in *B. burgdorferi* (Fig 1). Recent reports have described the contribution of intra-cellular acetate in the form of acetyl-phosphate in providing the activation signal to phosphorylate the response regulator Rrp2 which in turn leads to activation of the RpoN-RpoS regulatory pathway that facilitates adaptation by *B. burgdorferi* to vertebrate host-specific conditions [9], [37], [39]. An important determinant of this linear activation pathway is the balance in the levels of intracellular acetate, acetyl-phosphate and acetyl-CoA. Intracellular acetate is converted to acetyl-phosphate by the action of the enzyme acetate kinase (AckA) and in turn serves as a substrate for the enzyme phosphate acetyltransferase (Pta) which gives rise to acetyl-CoA. We and others have recently shown that increased levels of acetate transport and repression of Pta by the small RNA binding protein CsrA_Bb_ leads to increased levels of acetyl-phosphate which in turn leads to activation of the Rrp2-RpoN-RpoS pathway [9], [36], [39]. In this study, we determined the effect of intracellular acetate in not only activating the Rrp2-RpoN-RpoS pathway but also in its role for providing the initial substrate (acetyl-CoA) of the MP in *B. burgdorferi.* Moreover, we also determined the modulation of the levels of key enzymes (AckA and Pta) that are responsible for generating substrates that link intracellular acetate levels in activating gene regulatory networks as well as other metabolic pathways (such as the MP) that are operative in *B. burgdorferi* [67], [68]. Hence, the biochemical analysis of the MP links host-specific adaptation with vital physiological processes such as cell wall biogenesis and post-translational modifications in *B.* *burgdorferi*.

The ability of simvastatin and lovastatin to inhibit the HMGR activity indicated that the borrelial homolog is a bona fide HMG-CoA reductase, whose activity can be inhibited with micromolar concentrations of statins (Fig 3B). The effects of statins were also evident in the growth inhibition observed with *B. burgdorferi* treated with simvastatin or lovastatin (Fig. 9A-G). The inhibitory property of stains has several implications for interactions of *B. burgdorferi* with its hosts. For example, statins can directly inhibit the MP of *B. burgdorferi* and alter the cell wall biosynthesis or modulate the incorporation of cholesterol derivatives into glycolipids in vertebrate hosts that play a role in the transmission kinetics of the spirochetes and thereby have the potential to alter the incidence of Lyme disease. Moreover, the availability of a wide range of statins with different pharmacokinetics properties provide an attractive platform to develop formulations that can target the reservoir hosts to reduce their spirochetal burden and provide strategies to reduce the transmission of this tick-borne pathogen.

The adaptation of *B. burgdorferi* to highly disparate environmental signals present in the tick vector or vertebrate host has been extensively studied and a variety of signals such as temperature, pH, levels of dissolved gases, and other undefined host factors are known to rapidly alter gene expression to facilitate adaptation to the respective hosts [11], [40]–[45], [69]. In line with these observations, it was important to determine if the members of the MP are preferentially expressed under conditions that mimic the tick mid-gut before and after a blood meal. As shown in Fig 5, the levels of HMGR were up-regulated under fed-tick conditions (Fig. 5B, 5C, Lane 2) compared to unfed-tick conditions (Fig 5B, 5C Lane 1) while the other three downstream enzymes of the MP were up-regulated under the tick-specific conditions *B. burgdorferi*. It is possible that under fed-tick conditions, where the levels of acetyl-CoA are low due to repression of *pta* by CsrA_Bb_ (data not shown), an increase in the levels of HMGR, the rate limiting enzyme of MP, may be needed to generate sufficient levels of mevalonate while the levels of other enzymes under these conditions could be sufficient to obtain the end product, namely IPP. Since IPP is essential for peptidoglycan biosynthesis and the bacteria undergo more rapid growth following the ingestion of a blood meal by the ticks, it is logical that the rate-limiting enzyme in the IPP biosynthetic pathway would be upregulated under these specific conditions. It is also possible that the regulation of the members of the MP is different in response to alterations in pH and temperature, accounting for the differences in the levels of HMGR compared to other members of the MP. Nonetheless, the host-specific conditions do appear to alter the levels of the enzymes of the MP and probably coincide with the need for synchrony in regulating metabolic pathways consistent with host-specific adaptation.

Since acetyl-CoA is the initiating substrate, we further examined the effect of levels of acetate both on synthesis of the members of the MP as well as on regulators and determinants that are synthesized differentially under vertebrate host-specific conditions. Recent studies have shown that the increased transport of acetate into the bacterium allows for an increased upregulation of numerous mammalian-specific proteins [9], [39]. As acetate is normally funneled to produce acetyl-CoA, one of the starting components of the mevalonate pathway, it follows that increased acetate availability would lead to increased acetyl-CoA production, facilitating increased expression of proteins involved in MP. Consistent with this hypothesis, the levels of all the members of MP tested were increased in spirochetes propagated in media with 90 mM of supplemental acetate. This was true under conditions that mimicked the fed tick (Fig. 6B; pH 6.7/37°C), unfed tick (Fig. 8B; pH 7.6/23°C) or under laboratory growth conditions (Fig. 7B; pH 7.6/32°C).

Consistent with our previous report [9], the level of OppA5 was elevated in response to increasing levels of supplemental acetate. Interestingly, the increased levels of supplemental acetate (90 mM acetate) were capable of increasing the levels of RpoS and CsrA_Bb_, and therefore members of the *rpoS* regulon such as DbpA, BBK32 and OspC were elevated as well. It is not clear whether higher levels of regulators of vertebrate host-specific gene expression such as RpoS and CsrA_Bb_ or the higher levels of acetate alone are driving increased levels of OppA5. Regardless, OppA5 may contribute to the transport of acetate from the extracellular milieu and serve as a mediator connecting the external signals to adaptive gene expression in *B. burgdorferi.*

We and others have observed reduction in the levels of FlaB when *csrA_Bb_* was over-expressed *in trans* from a plasmid expressing *csrA_Bb_* under the control of constitutive borrelial promoter P*_flgB_* or using an IPTG inducible plasmid [10], [38], [70]. Since *csrA_Bb_* (*bb0184*) is the terminal ORF of a large *flgK* motility operon, it appears that the effect of increased levels of CsrA_Bb_ not synchronized with levels of other motility related genes may contribute directly or indirectly to the reduction in levels of FlaB. In this study, the increased levels of *csrA_Bb_* expressed from the native copy on the chromosome in response to increased levels of acetate do not appear to repress *flaB* or alter its synthesis presumably due to synchronized levels of other members of the *flgK* motility operon. We are also aware of the effect of levels of CsrA_Bb_ on other members of the *flgK* motility operon, which in turn, could affect the levels of FlaB directly or indirectly (Karna and Seshu, unpublished data).

A critical link to the levels of acetate and the regulators of vertebrate host-specific gene expression appears to be the level of acetyl-phosphate, as we observed increased levels of the enzyme acetate kinase (AckA) while there was a reduction in the levels of phosphate acetyltransferase (Pta), which is involved in converting acetyl-phosphate to acetyl-CoA (Fig. 1). It is important to note that increased acetate was able to modulate vertebrate host-specific adaptation even under environmental signals that are reflective of conditions present in the mid-gut of unfed ticks (pH 7.6/23°C; Fig. 8D-F), suggesting that minor modifications in the external signals could alter the response in *B. burgdorferi*. It can be argued that the levels of acetate used in this study are not physiological and that levels of these proteins may be partially reflective of what is observed under vertebrate host-specific conditions [9], [39]. It should be pointed out that the serum level of acetate in rodents is higher than the level of acetate in borrelial growth medium [39] and that this signaling mechanism does not incorporate other host-derived factors that could exert synergistic effects in addition to acetate in modulating the levels of these determinants.

In summary, we have shown that acetate serves as a key substrate that can modulate gene regulatory networks critical for vertebrate host-specific gene expression in *B. burgdorferi* and plays a central role in connecting metabolic pathways critical for growth and survival of *B. burgdorferi* with host-specific adaptation (Fig. 1). These studies have also added the contributions of extracellular factors such as acetate that may vary in concentration in different hosts as critical signals altering regulation of gene expression in *B. burgdorferi*. In conclusion, *B. burgdorferi* has a functional MP and acetate plays a critical role in modulating the vertebrate host-specific adaptation in conjunction with pathways that are essential for survival and growth of *B. burgdorferi*. These studies also helped identify pathways and proteins that could be targeted for inhibition in reservoir hosts, thereby expanding our ability to reduce the incidence of Lyme disease.

## References

[1] CDC (2011). Summary of notifiable diseases – United States, 2009.. Morb Mortal Wkly Rep.

[2] Barbour AG (1989). The molecular biology of Borrelia.. Rev Infect Dis.

[3] Orloski KA, Hayes EB, Campbell GL, Dennis DT (2000). Surveillance for Lyme disease–United States, 1992–1998.. MMWR CDC Surveill Summ.

[4] Barbour AG, Fish D (1993). The biological and social phenomenon of Lyme disease.. Science.

[5] Esteve-Gassent MD, Elliott NL, Seshu J (2009). sodA is essential for virulence of *Borrelia burgdorferi* in the murine model of Lyme disease.. Mol Microbiol.

[6] Karna SL, Sanjuan E, Esteve-Gassent MD, Miller CL, Maruskova M (2011). CsrA modulates levels of lipoproteins and key regulators of gene expression critical for pathogenic mechanisms of *Borrelia burgdorferi*.. Infect Immun.

[7] Maruskova M, Esteve-Gassent MD, Sexton VL, Seshu J (2008). Role of the BBA64 locus of *Borrelia burgdorferi* in early stages of infectivity in a murine model of Lyme disease.. Infect Immun.

[8] Maruskova M, Seshu J (2008). Deletion of BBA64, BBA65, and BBA66 loci does not alter the infectivity of *Borrelia burgdorferi* in the murine model of Lyme disease.. Infect Immun.

[9] Raju BV, Esteve-Gassent MD, Karna SL, Miller CL, Van Laar TA (2011). Oligopeptide permease A5 modulates vertebrate host-specific adaptation of *Borrelia burgdorferi*.. Infect Immun.

[10] Sanjuan E, Esteve-Gassent MD, Maruskova M, Seshu J (2009). Overexpression of CsrA (BB0184) alters the morphology and antigen profiles of *Borrelia burgdorferi*.. Infect Immun.

[11] Steere AC, Coburn J, Glickstein L (2004). The emergence of Lyme disease.. J Clin Invest.

[12] Bratton RL, Whiteside JW, Hovan MJ, Engle RL, Edwards FD (2008). Diagnosis and treatment of Lyme disease.. Mayo Clin Proc.

[13] Dandache P, Nadelman RB (2008). Erythema migrans.. Infect Dis Clin North Am 22: 235–260,.

[14] Nardelli DT, Munson EL, Callister SM, Schell RF (2009). Human Lyme disease vaccines: past and future concerns.. Future Microbiol.

[15] Takayama K, Rothenberg RJ, Barbour AG (1987). Absence of lipopolysaccharide in the Lyme disease spirochete, *Borrelia burgdorferi*.. Infect Immun.

[16] Fraser CM, Casjens S, Huang WM, Sutton GG, Clayton R (1997). Genomic sequence of a Lyme disease spirochaete, *Borrelia burgdorferi*.. Nature.

[17] Ben-Menachem G, Kubler-Kielb J, Coxon B, Yergey A, Schneerson R (2003). A newly discovered cholesteryl galactoside from *Borrelia burgdorferi*.. Proc Natl Acad Sci U S A.

[18] Schroder NW, Schombel U, Heine H, Gobel UB, Zahringer U (2003). Acylated cholesteryl galactoside as a novel immunogenic motif in *Borrelia burgdorferi* sensu stricto.. J Biol Chem.

[19] Stubs G, Fingerle V, Wilske B, Gobel UB, Zahringer U (2009). Acylated cholesteryl galactosides are specific antigens of borrelia causing lyme disease and frequently induce antibodies in late stages of disease.. J Biol Chem.

[20] LaRocca TJ, Crowley JT, Cusack BJ, Pathak P, Benach J (2010). Cholesterol lipids of *Borrelia burgdorferi* form lipid rafts and are required for the bactericidal activity of a complement-independent antibody.. Cell Host Microbe.

[21] Bochar DA, Stauffacher CV, Rodwell VW (1999). Sequence comparisons reveal two classes of 3-hydroxy-3-methylglutaryl coenzyme A reductase.. Mol Genet Metab.

[22] Hedl M, Rodwell VW (2004). Inhibition of the class II HMG-CoA reductase of *Pseudomonas mevalonii*.. Protein Sci.

[23] Theivagt AE, Amanti EN, Beresford NJ, Tabernero L, Friesen JA (2006). Characterization of an HMG-CoA reductase from *Listeria monocytogenes* that exhibits dual coenzyme specificity.. Biochemistry.

[24] Voynova NE, Rios SE, Miziorko HM (2004). *Staphylococcus aureus* mevalonate kinase: isolation and characterization of an enzyme of the isoprenoid biosynthetic pathway.. J Bacteriol.

[25] Wilding EI, Brown JR, Bryant AP, Chalker AF, Holmes DJ (2000). Identification, evolution, and essentiality of the mevalonate pathway for isopentenyl diphosphate biosynthesis in gram-positive cocci.. J Bacteriol.

[26] Wilding EI, Kim DY, Bryant AP, Gwynn MN, Lunsford RD (2000). Essentiality, expression, and characterization of the class II 3-hydroxy-3-methylglutaryl coenzyme A reductase of *Staphylococcus aureus*.. J Bacteriol.

[27] Lange BM, Rujan T, Martin W, Croteau R (2000). Isoprenoid biosynthesis: the evolution of two ancient and distinct pathways across genomes.. Proc Natl Acad Sci U S A.

[28] Wanke M, Skorupinska-Tudek K, Swiezewska E (2001). Isoprenoid biosynthesis via 1-deoxy-D-xylulose 5-phosphate/2-C-methyl-D-erythritol 4-phosphate (DOXP/MEP) pathway.. Acta Biochim Pol.

[29] Friesen JA, Rodwell VW (2004). The 3-hydroxy-3-methylglutaryl coenzyme-A (HMG-CoA) reductases.. Genome Biol.

[30] Istvan ES, Deisenhofer J (2001). Structural mechanism for statin inhibition of HMG-CoA reductase.. Science.

[31] Endo A (1992). The discovery and development of HMG-CoA reductase inhibitors.. J Lipid Res.

[32] Hamelin BA, Turgeon J (1998). Hydrophilicity/lipophilicity: relevance for the pharmacology and clinical effects of HMG-CoA reductase inhibitors.. Trends Pharmacol Sci.

[33] Tobert JA (2003). Lovastatin and beyond: the history of the HMG-CoA reductase inhibitors.. Nat Rev Drug Discov.

[34] Bellosta S, Paoletti R, Corsini A (2004). Safety of statins: focus on clinical pharmacokinetics and drug interactions.. Circulation.

[35] Bonetti PO, Lerman LO, Napoli C, Lerman A (2003). Statin effects beyond lipid lowering–are they clinically relevant?. Eur Heart J.

[36] Samuels DS (2011). Gene regulation in *Borrelia burgdorferi*.. Annu Rev Microbiol.

[37] CW, Li C (2011). Inactivation of bb0184, which encodes carbon storage regulator A, represses the infectivity of *Borrelia burgdorferi*.. Infect Immun.

[38] Sze CW, Morado DR, Liu J, Charon NW, Xu H (2011). Carbon storage regulator A (CsrA(Bb) ) is a repressor of *Borrelia burgdorferi* flagellin protein FlaB.. Mol Microbiol.

[39] Xu H, Caimano MJ, Lin T, He M, Radolf JD (2010). Role of acetyl-phosphate in activation of the Rrp2-RpoN-RpoS pathway in *Borrelia burgdorferi*.. PLoS Pathogens.

[40] Hubner A, Yang X, Nolen DM, Popova TG, Cabello FC (2001). Expression of *Borrelia burgdorferi* OspC and DbpA is controlled by a RpoN-RpoS regulatory pathway.. Proc Natl Acad Sci U S A.

[41] Lybecker MC, Samuels DS (2007). Temperature-induced regulation of RpoS by a small RNA in *Borrelia burgdorferi*.. Mol Microbiol.

[42] Rogers EA, Terekhova D, Zhang HM, Hovis KM, Schwartz I (2009). Rrp1, a cyclic-di-GMP-producing response regulator, is an important regulator of *Borrelia burgdorferi* core cellular functions.. Mol Microbiol.

[43] Schwan TG, Piesman J, Golde WT, Dolan MC, Rosa PA (1995). Induction of an outer surface protein on *Borrelia burgdorferi* during tick feeding.. Proc Natl Acad Sci U S A.

[44] Stevenson B, Schwan TG, Rosa PA (1995). Temperature-related differential expression of antigens in the Lyme disease spirochete, *Borrelia burgdorferi*.. Infect Immun.

[45] Yang XF, Alani SM, Norgard MV (2003). The response regulator Rrp2 is essential for the expression of major membrane lipoproteins in *Borrelia burgdorferi*.. Proc Natl Acad Sci U S A.

[46] Brissette CA, Verma A, Bowman A, Cooley AE, Stevenson B (2009). The *Borrelia burgdorferi* outer-surface protein ErpX binds mammalian laminin.. Microbiology.

[47] Jutras BL, Verma A, Adams CA, Brissette CA, Burns LH (2012). BpaB and EbfC DNA-binding proteins regulate production of the Lyme disease spirochete's infection-associated Erp surface proteins.. J Bacteriol.

[48] Seemanapalli SV, Xu Q, McShan K, Liang FT (2010). Outer surface protein C is a dissemination-facilitating factor of *Borrelia burgdorferi* during mammalian infection.. PLoS One.

[49] Labandeira-Rey M, Skare JT (2001). Decreased infectivity in *Borrelia burgdorferi* strain B31 is associated with loss of linear plasmid 25 or 28–1.. Infect Immun.

[50] Tilly K, Bestor A, Dulebohn DP, Rosa PA (2009). OspC-independent infection and dissemination by host-adapted *Borrelia burgdorferi*.. Infect Immun.

[51] Barbour AG (1984). Isolation and cultivation of Lyme disease spirochetes.. Yale J Biol Med.

[52] Robinzon S, Dafa-Berger A, Dyer MD, Paeper B, Proll SC (2009). Impaired cholesterol biosynthesis in a neuronal cell line persistently infected with measles virus.. J Virol.

[53] Ghosh PM, Mott GE, Ghosh-Choudhury N, Radnik RA, Stapleton ML (1997). Lovastatin induces apoptosis by inhibiting mitotic and post-mitotic events in cultured mesangial cells.. Biochim Biophys Acta.

[54] Seshu J, Boylan JA, Hyde JA, Swingle KL, Gherardini FC (2004). A conservative amino acid change alters the function of BosR, the redox regulator of *Borrelia burgdorferi*.. Mol Microbiol.

[55] Gilbert MA, Morton EA, Bundle SF, Samuels DS (2007). Artificial regulation of ospC expression in *Borrelia burgdorferi*.. Mol Microbiol.

[56] Lybecker MC, Abel CA, Feig AL, Samuels DS (2010). Identification and function of the RNA chaperone Hfq in the Lyme disease spirochete *Borrelia burgdorferi*.. Mol Microbiol.

[57] Seshu J, Esteve-Gassent MD, Labandeira-Rey M, Kim JH, Trzeciakowski JP (2006). Inactivation of the fibronectin-binding adhesin gene bbk32 significantly attenuates the infectivity potential of *Borrelia burgdorferi*.. Mol Microbiol.

[58] Samuels DS (1995). Electrotransformation of the spirochete *Borrelia burgdorferi*.. Methods Mol Biol.

[59] Liang K, Vaziri ND (2003). HMG-CoA reductase, cholesterol 7alpha-hydroxylase, LCAT, ACAT, LDL receptor, and SRB-1 in hereditary analbuminemia.. Kidney Int.

[60] Miziorko HM (2011). Enzymes of the mevalonate pathway of isoprenoid biosynthesis.. Arch Biochem Biophys.

[61] Takahashi S, Kuzuyama T, Seto H (1999). Purification, characterization, and cloning of a eubacterial 3-hydroxy-3-methylglutaryl coenzyme A reductase, a key enzyme involved in biosynthesis of terpenoids.. J Bacteriol.

[62] Akins DR, Bourell KW, Caimano MJ, Norgard MV, Radolf JD (1998). A new animal model for studying Lyme disease spirochetes in a mammalian host-adapted state.. J Clin Invest.

[63] Carroll JA, Garon CF, Schwan TG (1999). Effects of environmental pH on membrane proteins in *Borrelia burgdorferi*.. Infect Immun.

[64] Gilmore RD, Mbow ML, Stevenson B (2001). Analysis of *Borrelia burgdorferi* gene expression during life cycle phases of the tick vector *Ixodes scapularis*.. Microbe Infect.

[65] Ramamoorthy R, Scholl-Meeker D (2001). *Borrelia burgdorferi* proteins whose expression is similarly affected by culture temperature and pH.. Infect Immun.

[66] Schwan TG, Piesman J (2000). Temporal changes in outer surface proteins A and C of the lyme disease-associated spirochete, *Borrelia burgdorferi*, during the chain of infection in ticks and mice.. J Clin Microbiol.

[67] Sultan SZ, Pitzer JE, Boquoi T, Hobbs G, Miller MR (2011). Analysis of the HD-GYP domain cyclic dimeric GMP phosphodiesterase reveals a role in motility and the enzootic life cycle of *Borrelia burgdorferi*.. Infect Immun.

[68] Pitzer JE, Sultan SZ, Hayakawa Y, Hobbs G, Miller MR (2011). Analysis of the *Borrelia burgdorferi* cyclic-di-GMP-binding protein PlzA reveals a role in motility and virulence.. Infect Immun.

[69] Seshu J, Boylan JA, Gherardini FC, Skare JT (2004). Dissolved oxygen levels alter gene expression and antigen profiles in *Borrelia burgdorferi*.. Infect Immun.

[70] Mukherjee S, Yakhnin H, Kysela D, Sokoloski J, Babitzke P (2011). CsrA-FliW interaction governs flagellin homeostasis and a checkpoint on flagellar morphogenesis in *Bacillus subtilis*.. Mol Microbiol.

[71] Elias AF, Stewart PE, Grimm D, Caimano MJ, Eggers CH (2002). Clonal polymorphism of *Borrelia burgdorferi* strain B31 MI: implications for mutagenesis in an infectious strain background.. Infect Immun.

[72] Bischoff KM, Rodwell VW (1996). 3-Hydroxy-3-methylglutaryl-coenzyme A reductase from Haloferax volcanii: purification, characterization, and expression in *Escherichia coli*.. J Bacteriol.

[73] Bischoff KM, Rodwell VW (1992). Biosynthesis and characterization of (S)-and (R)-3-hydroxy-3-methylglutaryl coenzyme A. Biochem Med Meta Biol.

[74] Smythe CD, Greenall M, Kealey T (1998). The activity of HMG-CoA reductase and acetyl-CoA carboxylase in human apocrine sweat glands, sebaceous glands, and hair follicles is regulated by phosphorylation and by exogenous cholesterol.. The J Invest Derma.

